# Pickering emulsion droplet-based biomimetic microreactors for continuous flow cascade reactions

**DOI:** 10.1038/s41467-022-28100-6

**Published:** 2022-01-25

**Authors:** Ming Zhang, Rammile Ettelaie, Lianlian Dong, Xiaolong Li, Ting Li, Xiaoming Zhang, Bernard P. Binks, Hengquan Yang

**Affiliations:** 1 School of Chemistry and Chemical Engineering, Shanxi University, Taiyuan, 030006 China; 2 Food Colloids Group, School of Food Science and Nutrition, University of Leeds, Leeds, LS2 9JT UK; 3 Department of Chemistry, University of Hull, Hull, HU6 7RX UK; 4 Key Laboratory of Chemical Biology and Molecular Engineering of Ministry of Education, Institute of Molecular Science, Shanxi University, Taiyuan, 030006 China

**Keywords:** Heterogeneous catalysis, Catalyst synthesis

## Abstract

A continuous flow cascade of multi-step catalytic reactions is a cutting-edge concept to revolutionize stepwise catalytic synthesis yet is still challenging in practical applications. Herein, a method for practical one-pot cascade catalysis is developed by combining Pickering emulsions with continuous flow. Our method involves co-localization of different catalytically active sub-compartments within droplets of a Pickering emulsion yielding cell-like microreactors, which can be packed in a column reactor for continuous flow cascade catalysis. As exemplified by two chemo-enzymatic cascade reactions for the synthesis of chiral cyanohydrins and chiral ester, 5 − 420 fold enhancement in the catalysis efficiency and as high as 99% enantioselectivity were obtained even over a period of 80 − 240 h. The compartmentalization effect and enriching-reactant properties arising from the biomimetic microreactor are theoretically and experimentally identified as the key factors for boosting the catalysis efficiency and for regulating the kinetics of cascade catalysis.

## Abstract (web summary)

A continuous flow cascade of multi-step catalytic reactions would provide significant advantages in faster reaction times, waste reduction, and lowered step-count of syntheses, yet this ideal remains challenging in practical applications. Here the authors describe continuous flow cascade catalysis through co-localization of two catalytically active subcompartments within Pickering emulsion droplets.

## Introduction

A cascade of multiple catalytic reactions in a continuous flow manner is an important concept to upgrade chemical synthesis because of its noteworthy advantages such as shortened reaction time, reduced waste generation, and avoidance of intermediate separation^1–5^. The methods to realize this concept have been developed and include such techniques as physical mixing of catalytically different solid particles^6^, and multi-layered beds^7^. However, these methods are still incapable of precisely controlling cascade reactions at nanoscale levels, leading to relatively low cascade reaction efficiency. The co-localization of multiple catalysts within microreactors is emerging as an exciting way to realize this concept with high cascade reaction efficiency but continues to be a cutting-edge research topic in that there remain many critical challenges to overcome, especially in relation to practical application to continuous flow cascade reactions^8,9^. An ideal cascade catalytic microreactor should meet the following requirements: (i) spatial isolation of different catalytically active sites, while still ensuring their close proximity; (ii) favorable microenvironments able to channel intermediate reactants to the next catalytic sites avoiding random diffusion; (iii) multiple catalysts working synergistically, and (iv) enable a long-term continuous flow process or multiple recycling of catalysts.

The living cell is an ideal model for the design of cascade catalytic microreactors as it allows multiple biocatalytic transformations to proceed very efficiently^10^. The high efficiency is in part attributed to the unique multi-level compartmentalization in cells that enables them to sequester reactants/intermediates and spatially isolate incompatible species within different organelles (sub-compartments (SCs))^11^. Motivated by the approach of nature in its realization, extensive efforts have been made to synthesize various biomimetic microreactors for executing cascade reactions, such as lipid vesicles^12,13^, polymersomes^14–17^, microcapsules^18–27^, coacervate droplets^28,29^, and proteinosomes^30,31^. Although encouraging progress has been achieved, most of these existing biomimetic microreactors either require relatively complex fabrication procedures or lack a proper liquid medium inside their interior, leading to either difficulty in rationally controlling the interior structures or inability to enrich/sequester reactants^32^. Moreover, these microreactors suffer from relatively low mechanical robustness and accordingly fail to find potential scenarios for their practical use^33^.

Very recently, flow Pickering emulsions, a type of continuous flow catalytic method, was developed by our group^34,35^. Pickering emulsion droplets containing molecular catalysts or enzymes are filled in a column reactor for continuous flow reaction, without significant pressure drop in much the same manner as for solid beads in a fixed-bed reactor. Reactants flow through the microchannels, formed between the packed droplets, as part of the continuous oil phase. In spite of the great success in improving many catalytic reactions, this method is still incapable of dealing with cascade reactions because of the lack of a method to co-localize catalysts within the same droplet.

**Fig. 1:**
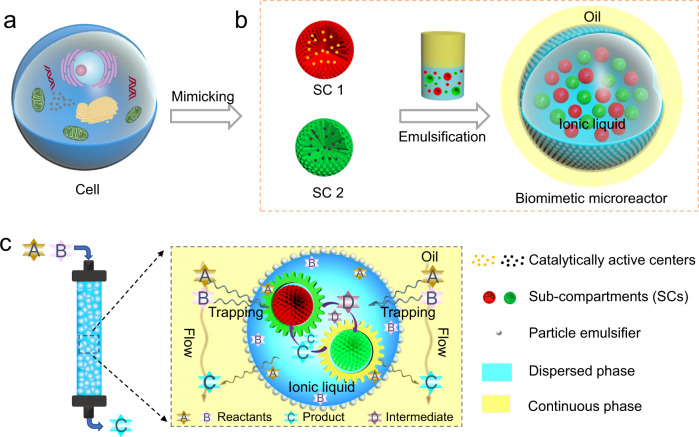
Schematics for the construction of biomimetic microreactors for continuous flow cascade reactions. **a** Microstructures in a living cell. **b** Co-localization of two distinct catalytically active sub-compartments (SCs) within a single droplet of ionic liquid-in-oil Pickering emulsion generating a biomimetic microreactor. **c** Column reactor packed with the biomimetic microreactors for continuous flow cascade reactions.

Herein, inspired by the multi-compartmental nature of living cells (Fig. 1a), we propose a Pickering emulsion strategy for the construction of a kind of “cell-like” microreactor, which is demonstrated to be capable of approaching practical efficient continuous flow cascade reactions. As Fig. 1b, c shows, our strategy involves co-localization of two different catalytically active SCs within a single liquid emulsion droplet via one-step emulsification, with a high level of control of the numbers of SCs as well as the ratio of different SCs (Fig. 1b, c). The obtained biomimetic microreactors are then packed in a fixed-bed reactor for continuous flow cascade catalysis, exhibiting 5- to 420-fold enhancement in catalysis efficiency (CE), 99% e.e. (enantiomeric excess) as well as durability of 240 h in continuous running. Furthermore, we theoretically and experimentally investigate how the compartmentalization effect and sequestering effect of the microreactors impact the CE. This work will provide a vast opportunity for practical cascade catalysis in view of the facile fabrication and structural flexibility of our biomimetic microreactor.

## Results

### Design and preparation of biomimetic microreactors

We propose a Pickering emulsion-based strategy to construct the biomimetic microreactors because Pickering emulsions (i.e., droplets stabilized by solid nanoparticles) are capable of compartmentalizing bulk liquid into nanoliter-scaled droplets.^36–39^ The droplets are proven to be more stable against coalescence in comparison to the commonly used surfactant-stabilized emulsions^40^, and can accordingly be harnessed as robust compartments to host catalytically active SCs. An ionic liquid (IL) is selected to act as the interior medium of the droplet microreactor because IL presents a more suitable medium for enriching certain organic compounds from other liquid phases^41^. Mesoporous silica nanoparticles (MSNs, diameter 80–90 nm) are chosen as SCs since the presence of nanopores in these particles is helpful for loading metal complex catalysts or enzymes (Supplementary Fig. 1 showing transmission electron microscopy (TEM) images, nitrogen sorption isotherms, and air–water contact angle of MSNs)^42^. A molecular catalyst (*S*, *S*)-{(salen)Ti-(µ-O)}_2_, abbreviated as Ti(Salen), and *Candida Antarctica* lipase B (CALB) were selected as a chemical catalyst and biocatalyst respectively (reasons for this choice are discussed later)^43^. Ti(Salen) and CALB were first supported separately on the mesopores of MSNs, yielding two catalytically active SCs, i.e., Ti/SCs (400 mg g^−1^ of Ti(Salen)) and CALB/SCs (72.3 mg g^−1^ of protein). The energy-dispersive X-ray spectroscopy elemental mapping shows that the elemental Ti (from Ti(Salen)) and N (from CALB) are homogeneously distributed on MSNs (Supplementary Fig. 2a, b). N_2_ sorption analysis also confirmed that Ti(Salen) and CALB entered the pores of MSNs because the pore sizes decreased from 8.0 nm down to either 5.0 nm or 6.7 nm (Supplementary Fig. 2c). Fourier transform infrared spectroscopy further confirms the successful loading of Ti(Salen) and CALB (Supplementary Fig. 2d). X-ray photoelectron spectroscopy results also revealed that there exist strong interactions between Ti(Salen) and MSNs (Supplementary Fig. 3).

**Fig. 2:**
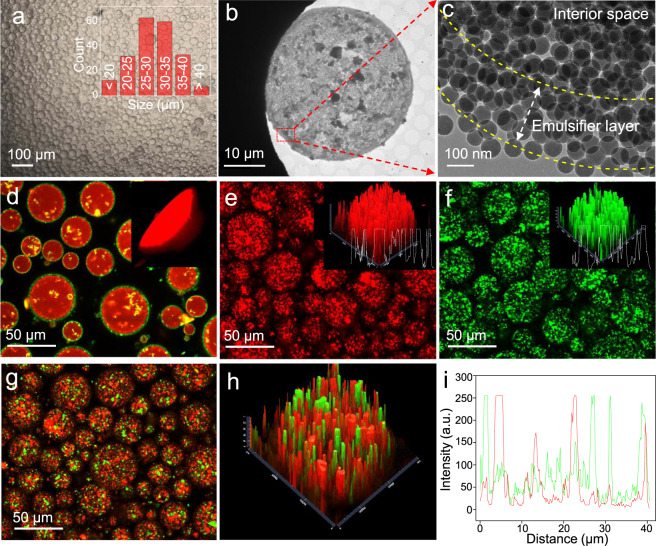
Structural characterization of the biomimetic microreactors. **a** Optical micrograph of an IL-in-oil Pickering emulsion-based microreactor housing SCs; the inset showing the droplet size distribution. **b** Cryo-TEM image of a frozen SCs-containing water droplet in oil. **c** Magnified cryo-TEM image of (**b**) showing the interface of the SCs-containing microreactor. **d** Confocal laser scanning microscopy image of the microreactor within which SCs were dyed with Rhodamine B and the solid particle emulsifier was dyed with FITC-I; inset shows its 3D image. **e** Confocal laser scanning microscopy image of the microreactors within which Ti/SCs were dyed with Rhodamine B (red). **f** Confocal laser scanning microscopy image of the microreactors within which CALB/SCs were dyed with FITC-I (green); 3D image shows the distribution of SCs within the microreactor and the fluorescence intensity profile along its diameter. **g** Confocal laser scanning microscopy image of the biomimetic microreactor containing FITC-I-labeled SCs and Rhodamine B-labeled SCs. **h** 3D confocal laser scanning microscopy image for the biomimetic microreactor in (**g**). **i** Fluorescence intensity profiles showing the distribution of the two different fluorescently labeled SCs.

The fabrication of biomimetic microreactors involves compartmentalization of SCs within IL droplets by one-step emulsification of a biphasic mixture of [BMIM]PF_6_ (IL), *n*-octane, and SCs using hydrophobic silica nanospheres (60–80 nm in diameter) as emulsifiers (Supplementary Figs. 4 and 5). The  obtained IL-in-oil droplet microreactors were confirmed by the fluorescence dyeing experiment (Supplementary Fig. 6). Due to the high hydrophilicity of SCs (being more hydrophilic than the emulsifier particles as evidenced by air–water contact angle measurements), they should be distributed within the IL droplets since SCs prefer to reside in the IL phase before emulsification. Before examining the co-localization of two kinds of SCs, we checked the feasibility of the compartmentalization of one kind of SCs. Figure 2a shows an optical micrograph of SCs-containing droplets which exhibit a spherical morphology and the size distribution was centered at 30 ± 10 µm. The loading of SCs within the droplets can be adjusted by varying the amount of SCs added before emulsification. When the loading of SCs exceeds 20 wt% (with respect to the weight of IL), the droplets become non-spherical (Supplementary Fig. 7). This can be explained in terms of the interfacial jamming phenomenon^44^, where a small portion of SCs occupy the interface to prevent the relaxation of the droplet shape back to its spherical form during emulsification. The localization of SCs within the droplet was confirmed by cryo-TEM. Since the IL cannot be frozen, water was used here instead as the dispersed phase. A micron-sized sphere was clearly observed after freezing, originating from the emulsion droplet (Fig. 2b). In the magnified TEM images (Fig. 2c and Supplementary Fig. 8), silica particles of the emulsifier (i.e., the ones without nanopores) were seen to be closely packed on the surface of the microsphere, while SCs (i.e., the ones with nanopores) were relatively homogeneously distributed within the interior of the microsphere. This is consistent with fluorescence microscopy, showing a green fluorescent ring around the droplet and red fluorescent signals throughout the interior of the droplet (the emulsifier particles and SCs were labeled with fluorescein isothiocyanate I (FITC-I) and Rhodamine B respectively, Fig. 2d). To further confirm the distribution of the catalytically active SCs within microreactors, we labeled Ti/SCs with Rhodamine B and CALB/SCs with FITC-I, respectively. The confocal laser scanning microscope images revealed a homogenous distribution of Ti/SCs or CALB/SCs throughout the whole body of the droplets, as red fluorescence (from Rhodamine B-labeled SCs) or green fluorescence (from FITC-I-labeled SCs) was clearly detected (Fig. 2e, f). Both fluorescence intensities were found to be relatively even along the diameter of the droplets, once again verifying a reasonably homogeneous distribution of SCs within droplets. At the same time, no fluorescence signals were observed outside the droplets even if the SCs-loaded emulsions were shaken violently (Supplementary Fig. 9). This indicates a very high level of encapsulation efficiency and a good entrapment ability of the IL droplets toward the SCs. Such a good entrapment ability was further confirmed by a continuous flow experiment where the fluorescently labeled SCs were observed to remain within the droplets after 24 h of washing a microreactor-packed column with an oil phase (Supplementary Fig. 10). The ability to entrap SCs is attributed to the interfacial layers formed by the close packing of the emulsifier particles, thus providing a physical barrier to SCs escaping from droplets. Notably, even after standing for 48 h, these SCs remained relatively well dispersed (without severe aggregation) inside the droplets (Supplementary Fig. 11). In contrast, when the dispersed phase was replaced with water, SCs were observed to migrate to the interface of the droplets (Supplementary Fig. 12). The unique charge properties of IL may prevent the aggregation of negatively charged SCs^45^. The successful co-localization of Ti/SCs and CALB/SCs was also confirmed by a fluorescence experiment. After simultaneously introducing these two different fluorescently labeled SCs into a single droplet, their fluorescence intensities along the diameter of droplets were found to be both relatively even, also indicating a relatively homogeneous distribution of these two SCs within the same droplet (Fig. 2g–i), even after 48 h of actual continuous flow reaction (Supplementary Fig. 13). These results demonstrate that our judicious combination of a Pickering emulsion, IL and MSNs allows us to straightforwardly co-localize catalytically active SCs within a small microdomain, akin to the organization of organelles within living cells.

### Enhanced CE in the continuous flow system

**Fig. 3:**
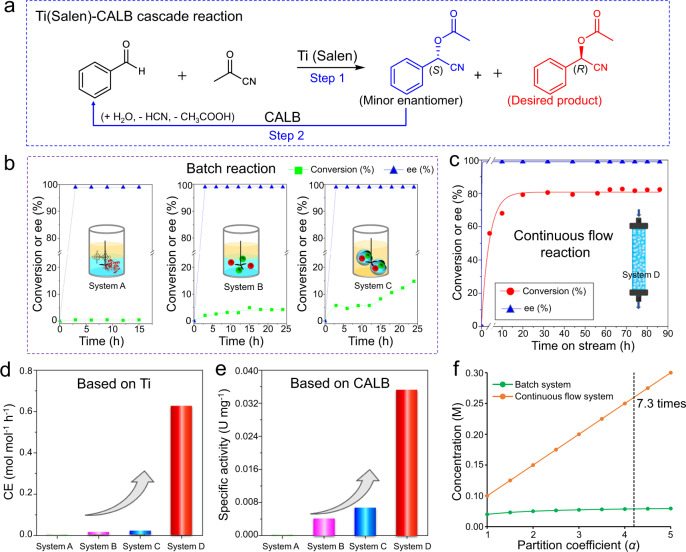
Chemo-enzymatic cascade reactions in batch and flow systems. **a** Cascade reaction for the synthesis of chiral O-acylated cyanohydrin by cascading Ti(Salen) and CALB. **b** Kinetic profiles for different batch cascade reactions. System A: using Ti(Salen) and CALB as catalysts; System B: Ti/SCs and CALB/SCs; System C: co-localization of Ti/SCs and CALB/SCs within a single microreactor (IL-in-oil emulsion). **c** Benzaldehyde conversion and e.e. of O-acylated cyanohydrin with time in the flow system with an IL-in-oil microreactor. **d** Catalysis efficiency in the batch and flow reactions. **e** Specific activity of CALB in different systems. For batch reactions, CE and specific activity were calculated according to the conversion within the first 10 h; for the flow reactions, CE was calculated after the conversion leveled off. **f** Calculated benzaldehyde concentration in IL as a function of the partition coefficient in the batch and flow systems. Batch reaction conditions: System A: 2.1 mL [BMIM]PF_6_, 3 mL PEG-300, 0.9 mL PBS (pH 7.4, 0.05 M), 0.08 g Ti(Salen), 0.03 g 4-dimethylaminopyridine (DMAP, as co-catalyst)^43^, 0.9 mL CALB (8.0 mg mL^−1^ of protein), 3 mL *n*-octane containing benzaldehyde (0.05 M) and acetyl cyanide (0.2 M), 25 °C, 900 rpm; System B: using Ti/SCs and CALB/SCs;System C: Ti/SCs and CALB/SCs co-localized within a single microreactor. Flow reaction conditions: the system consists of 2.1 mL [BMIM]PF_6_, 3 mL PEG-300, 0.9 mL PBS (pH 7.4, 0.05 M), 0.2 g Ti/SCs, 0.1 g CALB/SCs, 3 mL *n*-octane, 0.18 g emulsifier. Benzaldehyde (0.05 M) and acetyl cyanide (0. 2 M) in *n*-octane as mobile phase, 25 °C, flow rate = 1 mL h^−1^. All the reactions were repeated twice to obtain an average.

Next, we examined the catalytic performance of our biomimetic microreactors in the synthesis of chiral O-acylated cyanohydrins, a key chemical intermediate^46,47^. Ti(Salen) was reported to be capable of catalyzing asymmetric addition of acetyl cyanide to aldehydes yielding chiral O-acylated cyanohydrins but often suffered from a relatively low enantioselectivity^48^. To address this issue, Wingstrand and coworkers added CALB into a Ti(Salen)-involved reaction system to improve the enantioselectivity by hydrolyzing a minor, unwanted enantiomer of O-acylated cyanohydrins back to the starting material^43^. Despite a significant improvement of the enantioselectivity, the reaction efficiency was unsatisfactory in that a period of 144 h was required to complete the cascade reaction. The low efficiency may be attributed to the incompatibility between Ti(Salen) and CALB to some extent. We reason that if these two catalyst-containing SCs are co-localized in the biomimetic microreactor, Ti(Salen) and CALB can physically remain close but yet be isolated from each other. Within the microreactor, the undesired minor enantiomer generated in the first reaction would subsequently be hydrolyzed back to the initial reactant in the presence of CALB. After multiple cycles, all the starting reactants could eventually be converted to the desired enantiomer (Fig. 3a).

Before testing continuous flow reactions, we carried out a set of batch reactions to identify the advantages of the co-localization of two catalytically active SCs within a single microreactor. First, a traditionally biphasic reaction consisting of *n*-octane, [BMIM]PF_6_, PEG-300, and a small amount of water in the presence of Ti(Salen) and CALB, was examined. The addition of PEG-300 here serves to help dissolve water (as a reactant) in [BMIM]PF_6_. After 10 h of reaction, the benzaldehyde conversion was only 0.5% (Fig. 3b, system A), although 99% e.e. of O-acylated cyanohydrin was obtained. When Ti(Salen) and CALB were replaced with Ti/SCs and CALB/SCs, the conversion was improved to 5.6% under the same conditions (Fig. 3b, system B), indicating that the spatial isolation of these two catalysts is necessary for this one-pot cascade reaction. Remarkably, when using our designed biomimetic microreactors (IL-in-oil emulsion) in which both Ti/SCs and CALB/SCs were simultaneously present, the conversion was further increased up to 15% (Fig. 3b, system C), which is 3.3 times higher than that in the biphasic system highlighting the advantage of the use of biomimetic microreactors.

To further improve the reaction efficiency, we turned to the Pickering emulsion droplet-based continuous flow system (as supplementary Fig. 14 shows, the filling of solid particles in the droplets does not affect the flow rate and droplet morphology). A solution of the reactants was continuously pumped into the inlet of the column reactor and the product-rich stream was collected from its outlet. The benzaldehyde conversion gradually rose up to 81% and the e.e. for O-acylated cyanohydrin was 99% (Fig. 3c). On the basis of the conversions obtained in batch and flow systems, we estimated their CE (defined as the moles of converted reactant per mole of Ti per h, i.e., mol mol^−1^ h^−1^, Fig. 3d) based on Ti and the specific activity of CALB (expressed as μmol of substrate converted per min per mg enzyme, i.e., U mg^−1^, Fig. 3e). The continuous flow system gave a CE of 0.61 mol mol^−1^ h^−1^ which is about 14 times higher than that of the biomimetic microreactors in the batch reaction (Fig. 3d, system C, 0.043 mol mol^−1^ h^−1^), 30 times higher than that of Ti/SCs and CALB/SCs in the batch system without compartmentalization (system B, 0.002 mol mol^−1^ h^−1^) and 420 times higher than that of the direct mixing of Ti(Salen) and CALB (system A, 1.5 × 10^−4^ mol mol^−1^ h^−1^). Meanwhile, the specific activity of CALB in the continuous flow system was 0.035 U mg^−1^, exhibiting a 5.4, 8.8, and 194.4-fold enhancement relative to systems C, B, and A (Fig. 3e).

### Reactant sequestering effect in the microreactor-based continuous flow system

In order to understand the reasons for the enhanced CE in the Pickering emulsion microreactor, we established a theoretical model to analyze the CE in the biomimetic microreactor-based batch and continuous flow systems. We consider a general set of cascade reactions, where the first step of the reaction converts the reagent A to either C or D (with C and D being the enantiomer forms of each other) through a reaction A + B ⇋ 3/5 C + 2/5 D. The probability of conversion to C was 3/5 and to D 2/5, in accordance with the chiral preference of Ti(Salen) in our actual experiments. The second step, converting D back to A, involves D + E ⇋ A + F. The maximum theoretically achievable conversion in a batch system occurs when the system attains equilibrium. The resulting conversion can be shown to be (detailed derivation in the Supplementary Information)

1 $${{{{{\rm{Conversion}}}}}}_{{{{{\rm{batch}}}}}}=1-{\left[\begin{array}{c}1+\left({k}_{1}\frac{{\alpha }_{A}{\alpha }_{B}({\alpha }_{C}\phi +(1-\phi ))}{{\alpha }_{C}({\alpha }_{A}\phi +(1-\phi ))({\alpha }_{B}\phi +(1-\phi ))}\right)\\ \left(\frac{1}{{k}_{2}}+\frac{1}{{k}_{2}+(2.5{k}_{3}{\alpha }_{E}(1-\phi ){E}_{o}^{in})/({\alpha }_{E}\phi +(1-\phi ))}(1-\phi ){B}_{o}^{in}\right)\end{array}\right]}^{-1}$$

where *ϕ* is the volume fraction of the dispersed IL phase and ${B}_{o}^{{in}}$ and ${E}_{o}^{{in}}$ the initial concentrations of reactants B and E in the oil, respectively. The amount of B and E are assumed here to be well in excess, which is consistent with the experiments. The parameters *k*_1_ and *k*_2_ are the forward and backward reaction rate constants for the first step of the cascade reaction, and *k*_3_ is the forward rate constant for the second step (the backward reaction rate constant *k*_4_ of the second step can safely be ignored, as explained in Supplementary Information). Eq. (1) also involves the partition coefficient, i.e., [reagent]_IL_/[reagent]_oil_, for reactants A, B, and E and products C or D, denoted here as *α*_*A*_, *α*_*B*_, *α*_*E*_, and *α*_*C*_, *α*_*D*_, respectively (*α*_*D*_ can be replaced by *α*_*C*_ since for enantiomer forms *α*_*C*_ = *α*_*D*_).

For the flow system under the steady-state conditions, the concentration of substances inside the IL attains constant values. Again, it is possible to calculate the maximum theoretically attainable conversion under such conditions. This, for a sufficiently long column, can be shown (Supplementary Information) to be

2 $${{{{{\rm{Conversion}}}}}}_{{{{{\rm{flow}}}}}}=1-{\left[1+\left({k}_{1}\frac{{\alpha }_{A}{\alpha }_{B}}{{\alpha }_{C}}\right)\left(\frac{1}{{k}_{2}}+\frac{1}{{k}_{2}+2.5{k}_{3}{\alpha }_{E}{E}_{o}^{in}}\right){B}_{o}^{in}\right]}^{-1}$$

where various symbols are the same as those defined previously for Eq. (1).

Comparing Eqs. (1) and (2), we find that the main difference between the two equations is the coefficient multiplying *k*_1_ in the second term in the square bracket. The ratio of this coefficient between the continuous flow and batch system is:

3 $$\frac{({\alpha }_{A}\phi +(1-\phi ))({\alpha }_{B}\phi +(1-\phi ))}{({\alpha }_{C}\phi +(1-\phi ))(1-\phi )}$$

Obviously, when the value of the above ratio is larger than 1, a higher conversion will be predicted for the continuous flow system. In the case of our reactions, we have *α*_*A*_ = 4.2, *α*_*B*_ = 28.0, and *α*_*C*_ = 26.0 (Supplementary Fig. 15) and *ϕ* = 0.6, which then implies that this ratio is 6.25 >> 1. Thus, a higher CE is achievable in the continuous flow system. This analysis illustrates that the difference in CE for these two systems results from better utilization of the reactant-enriching effect of IL in the continuous flow system, which is supported by the results from the experiments with varying the reactant concentrations in both batch and continuous flow systems (Supplementary Fig. 16). This enriching effect can be further demonstrated by a set of theoretical results through varying the partition coefficient *α* (Fig. 3f). Take reactant A as an example, for the batch system at time *t* = 0, the local concentration of *A* inside the droplets is given by $\frac{(1-\phi ){\alpha }_{A}}{{\alpha }_{A}\phi +(1-\phi )}{A}_{o}^{in}$. The corresponding concentration in the droplets at the top of the column at steady-state is ${{\alpha }_{A}A}_{o}^{{in}}$, which is some 7.3 times higher than the batch system in the case of *α*_*A*_ = 4.2. Due to a large partition coefficient of reactants in the IL phase *cf*. oil (for a given fixed *ϕ*), the continuous flow system enables the microreactors to continuously sequester reactants from the flowing oil phase, therefore obtaining a much higher local concentration of reactants than the batch system. In turn, these high concentrations of reactants within the microreactors promote high CE. These results deliver one feature of the biomimetic microreactor.

### Compartmentalization effects

**Fig. 4:**
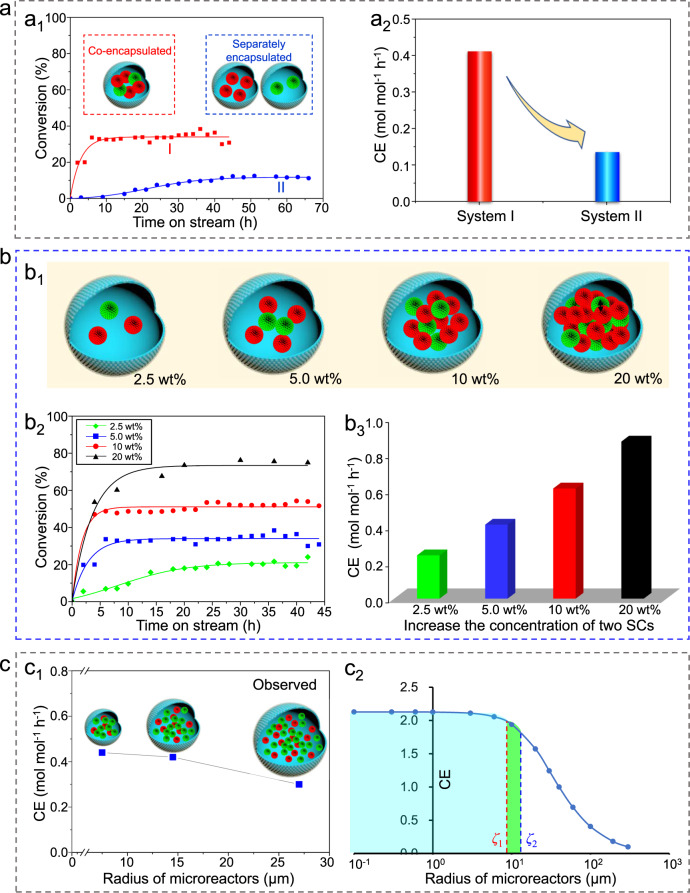
Compartmentalization effects. **a** Impact of compartmental manner. **a**_**1**_ Conversion with time for the chemo-enzymatic cascade flow reactions. The system I: two SCs co-localized within the same microreactor; system II: two SCs separately localized in different microreactors. **a**_**2**_ CE of the system I and system II. **b** Impact of SCs concentrations within microreactor. **b**_**1**_ Schematic illustration of the microreactor with varied local SCs concentrations but with fixed total catalyst amount and catalyst ratio (Ti/SCs:CALB/SCs is 2:1). **b**_**2**_ Conversion with time for the microreactors with different local SCs concentrations. **b**_**3**_ CE as a function of SCs concentration. **c** Impact of microreactor size. **c**_**1**_ CE vs. microreactor radius. **c**_**2**_ Theoretically predicted CE for microreactor with different radii. *ζ* is the distance away from the microreactor surface. All of the reaction conditions are the same as those in Fig. 3c except for the flow rate (2 mL h^−1^ here).

The other feature of the biomimetic microreactor is the compartmentalization effect, which significantly impacts the CE as verified by two sets of comparative experiments. One set of experiments was conducted where Ti/SCs and CALB/SCs were confined in the same microreactor or separately confined in two microreactors (Fig. 4a). Remarkably, the conversion for the case of co-localization at steady-state was 34.2% (Fig. 4a_1_), much higher than that of the case of separation (11%), i.e., a 3-fold increase in CE (Fig. 4a_2_). The other set of experiments was designed to compare the CE of the single-step reaction and the cascade reaction. For the single-step reaction of asymmetric addition of acetyl cyanide to aldehydes using Ti/SCs as a sole catalyst, Ti/SCs were either uniformly confined in all of the droplets or confined in only half of them (i.e., doubling the local catalyst concentration) with the other half being empty droplets devoid of Ti/SCs (Supplementary Fig. 17a). For the system with Ti/SCs present in all of the droplets, the benzaldehyde conversion at steady-state was 85.0% (Supplementary Fig. 17b), being more or less equal to the system where the SCs were confined only to half of the droplets (87.7%). The CE for these two systems was estimated to be 0.55 and 0.57 mol mol^−1^ h^−1^, respectively (Supplementary Fig. 17c). These findings demonstrate that changing the concentration of Ti/SCs does not affect the CE in a single-step reaction, provided that the droplets are chosen to be sufficiently small—see later for the required size. However, for the cascade reaction (Supplementary Fig. 18a), the reaction results are quite different. For the cascade system with Ti/SCs and CALB/SCs included in all of the droplets the benzaldehyde conversion at steady-state was 34.2% (Supplementary Fig. 18b). This is much lower than that of the system where all the catalysts were confined to only half of the droplets (72.9%, Supplementary Fig. 18b). The CE values are 0.42 and 0.89 mol mol^−1^ h^−1^, respectively (Supplementary Fig. 18c). The differences revealed by these two sets of experiments suggest that the co-localization of two SCs within the same microreactor is definitely favorable to promote the cascade reaction. The enhanced CE for the co-localization is due to the fact that the intermediate produced over Ti/SCs can diffuse directly and rapidly to CALB/SCs within the same microreactor (without escaping), thus accelerating the overall cascade reaction.

### Local catalyst concentration-dependent compartmentalization effect

The benefits of the catalyst co-localization inspired us to investigate the impact of the local catalyst concentration on CE. As shown in Fig. 4b_1_, when the total SCs concentration was varied from 2.5 to 5.0, 10, and 20 wt% (fixing the ratio of the two SCs at 2:1 and the total dosage), the benzaldehyde conversion at steady-state increased progressively from 20.2 to 34.2, 52.4, and 75.1% (Fig. 4b_2_) and the corresponding CE increased from 0.28 to 0.42, 0.61 and 0.83 mol mol^−1^ h^−1^ (Fig. 4b_3_). It is amazing that even though the increase in the SCs concentration led to a lower molecular diffusion rate (Supplementary Fig. 19), the remarkable increase in CE with local SCs concentration was still present. This local concentration-dependent CE again suggests that there exists a synergistic effect between the two catalytically active SCs within the same microreactor, which probably arises from their close proximity. The higher local catalyst concentrations correspond to shorter distances between the two SCs, which allows the intermediate to be rapidly converted in the subsequent reaction.

### Microreactor size-dependent compartmentalization effect

To further elaborate the compartmentalization effect, we examined microreactors with different droplet sizes (keeping the ratio of the two SCs and their total amount constant). Upon decreasing the microreactor droplet radius from 27 to 14.5 and 7.5 µm (Supplementary Fig. 20a), the conversion at steady-state increased progressively from 24.4 to 34.2 and 35.5% (Supplementary Fig. 20b) and the CE increased from 0.30 to 0.42 and 0.44 mol mol^−1^ h^−1^ (Fig. 4c_1_). To rationalize these results, we also established a theoretical model on the basis of two key parameters: the catalytic reaction rate and the reactant diffusion rate (presented in detail in the Supplementary Information). The main equation resulting from this model, for the rate of production of compound C in a droplet of size *R*, is as follows:

4 $${\gamma }_{C}= 	\, {a}_{2}Z\left(\frac{{d}_{f}}{{d}_{f}^{o}}\right)\left(\frac{R}{{\zeta }_{2}}\,\cosh (R/{\zeta }_{2})-\,\sinh (R/{\zeta }_{2})\right)-{a}_{1}Z\left(\frac{{d}_{f}}{{d}_{f}^{o}}\right)\\ 	 \,\left(\frac{R}{{\zeta }_{1}}\,\cosh (R/{\zeta }_{1})-\,\sinh (R/{\zeta }_{1})\right)$$

where *γ*_*C*_ is the rate of the generation of the product C, involved in a cascade reaction with two steps A + B ⇋ C + D and D + E ⇋ A + F, the same as the one discussed before. A very important feature of this equation is the emergence of two length scales *ζ*_1_ and *ζ*_2_ corresponding to the two steps of the reaction. Roughly speaking, *ζ* means the depth of reaction occurring (namely the distance away from the surface into the microreactor) beyond which a portion of catalysts are idle because of the excess of catalyst within microreactors. Beyond this distance any product molecule will find it difficult to diffuse out of the droplet prior to its conversion back to reactants, occurring as a result of backward reactions. If the reactant diffusion is fast and the catalytic reaction is slow, *ζ* will be great. The other parameters *Y*, *Z*, *a*_1_, *a*_2_, *d*_*f*_, and *d*_*f*_^*o*^ are defined in the Supplementary Information.

The above equation allows us to predict a theoretical value for CE for a given microreactor size (Fig. 4c_2_). It is interesting to find that the trend of the predicted theoretical values is reasonably close to the experimentally determined one. In the case of a local catalyst concentration of 5.0 wt%, the above two length scales *ζ*_1_ and *ζ*_2_ can be calculated to 9.2 and 26.3 µm according to Eqs. (35a) and (35b) in the Supplementary Information. In Fig. 4c_1_, for the region where the radius *R* (7.5 µm) is less than *ζ*_1_ and *ζ*_2_, the catalysts in the microreactors are fully utilized, and accordingly, the CE does not change significantly with the radius. When the radius *R* is 14.5 µm which is between *ζ*_1_ and *ζ*_2_, the CE begins to decrease because a portion of the catalysts is not fully utilized. For the region where the radius *R* (27.0 µm) is greater than both *ζ*_1_ and *ζ*_2_, even more catalysts are idle for both steps of the cascade reaction near the center of the microreactors leading to a greater decrease in the CE. These results not only support the fact that the developed biomimetic microreactor provides compartmentalization effects with a tunable impact on the cascade CE, but also allow us to predict the reaction progressing inside the microreactor in terms of measurable parameters.

### Local catalyst ratio-dependent compartmentalization effect

**Fig. 5:**
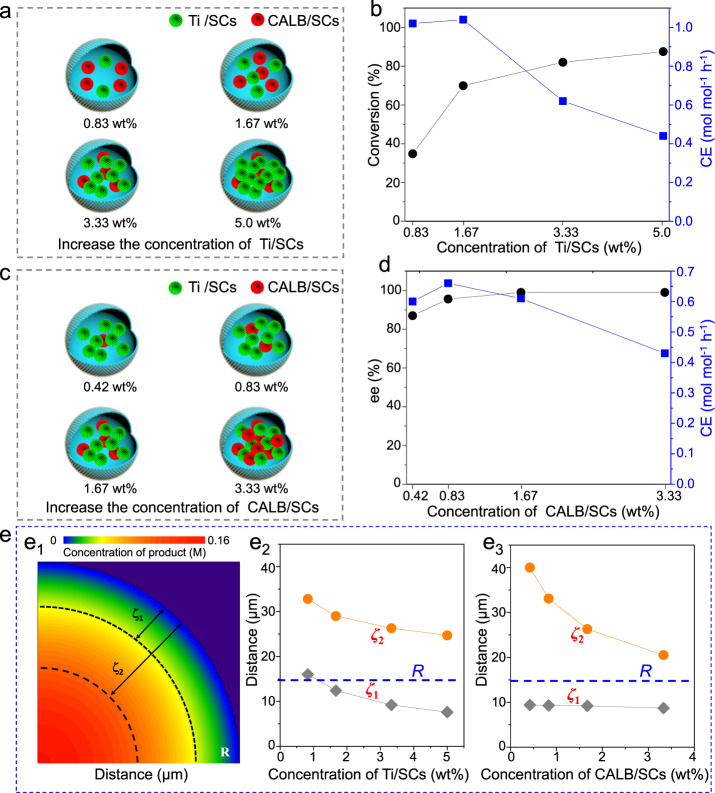
Catalysis efficiency for the microreactors with different catalyst ratios. **a** Schematic illustration of biomimetic microreactors with different fractions of Ti/SCs. **b** Benzaldehyde conversion and CE as a function of the fraction of Ti/SCs. **c** Schematic illustration of the biomimetic microreactors with different fractions of CALB/SCs. **d** E.e. and CE a function of the fraction of CALB/SCs. **e**_**1**_ Schematic illustration of the relationship between *ζ*_1_, *ζ*_2_, and *R*. **e**_**2**_ *ζ* as a function of the Ti/SCs concentration. **e**_**3**_ *ζ* as a function of the CALB/SCs concentration. The reaction conditions are the same as those in Fig. 3c except for the ratio of catalysts.

Next, we were curious as to the impact of the local catalyst ratio on CE. Two sets of biomimetic microreactors varying the ratio of SCs were examined separately (Fig. 5, all the reaction systems show similar morphologies and comparable droplet sizes in Supplementary Fig. 21). It was found that the conversions and e.e. were dependent on the ratio even under the same reaction conditions (Supplementary Figs. 22 and 23). In the first case where the concentration of Ti/SCs was varied from 0.83 to 5.0 wt%, while the concentration of CALB/SCs was kept at 1.67 wt% (Fig. 5a), the CE was calculated to be 1.02, 1.04, 0.61, and 0.44 mol mol^−1^ h^−1^, giving a maximum CE at 1.67 wt% loading of Ti/SCs (Fig. 5b). More interestingly, although the amount of enzyme is kept constant, its specific activity is also changed from 0.032 to 0.064, 0.076, and 0.080 U mg^−1^. Similarly, for the other case where the concentration of CALB/SCs was varied from 0.42 to 3.33 wt%, while the concentration of Ti/SCs was kept at 3.33 wt% (Fig. 5c), e.e. of the product increased with an increase in the concentration of CALB/SCs (e.e. values were 87.0, 95.6, 99.0, and 99.0%, Fig. 5d). Interestingly again, the CE was calculated to be 0.60, 0.66, 0.61, and 0.43 mol mol^−1^ h^−1^, first increasing and then decreasing despite keeping a constant concentration. These findings further point out the fact that the ratio of the two SCs affects the overall reaction kinetics within the microreactors.

To rationalize these results, the relationship between *ζ*, *R*, and the ratio of the two SCs was investigated (Fig. 5e_1_). As the Ti/SCs concentration was increased while keeping the concentration of CALB/SCs constant, both of *ζ*_1_ and *ζ*_2_ became progressively smaller (from 16.0 to 12.4, 9.2, and 7.6 µm; and 32.8 to 29.0, 26.3, and 24.7 µm, Fig. 5e_2_). Notably, *ζ*_2_ is always larger than the radius *R* (14.5 µm) and accordingly has little effect on the CE. However, *ζ*_1_ is first larger than *R*, then roughly the same as *R*, and finally less than *R* upon changing the Ti/SCs concentration. Therefore, the reaction efficiency first increased and then decreased. Similarly, upon increasing in the CALB/SCs concentration from 0.42 to 3.33 wt% (with the identical Ti/SCs concentration, Fig. 5e_3_), *ζ*_2_ is calculated to change from 40.0 to 20.5 µm which is much larger than *R* (14.5 µm), and *ζ*_1_ (from 9.4 to 8.7 µm) is always less than *R*. Consequently, the CE decreases as *ζ*_1_ becomes increasingly smaller with a larger concentration of CALB/SCs.

### Substrate scope, durability, and cascade reaction extension

**Fig. 6:**
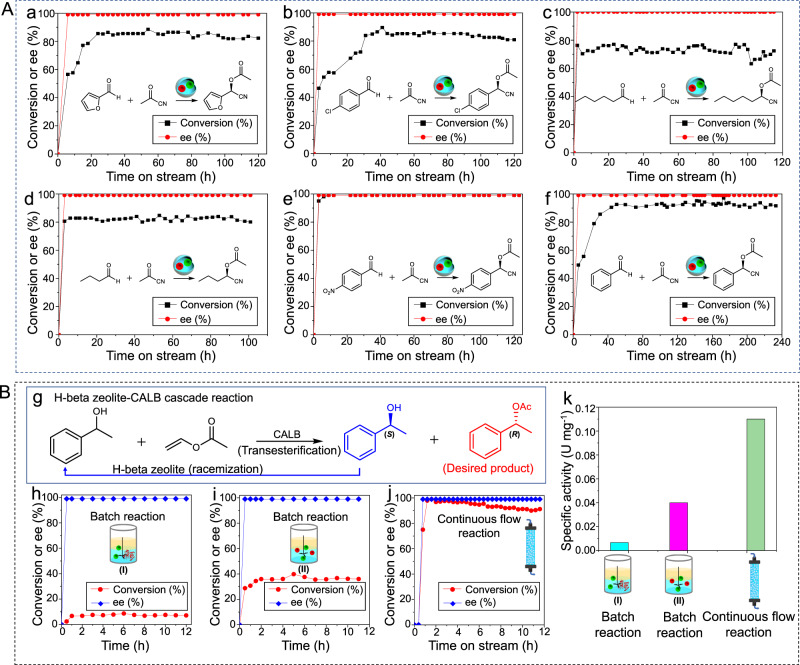
Substrate scope and cascade reaction extension. **A** Different substrates for Ti(Salen)-CALB cascade flow reactions. The system consists of 0.2 g Ti/SCs, 0.1 g CALB/SCs, 2.1 mL [BMIM]PF_6_, 3 mL PEG-300, and 0.9 mL PBS (pH = 7.4, 0.05 M), 3 mL *n*-octane, 0.18 g emulsifier. Reaction conditions: aldehyde (0.05 M) and acetyl cyanide (0.2 M) in *n*-octane, 25 °C. **a** Flow rate from initial 0.6–0.3 mL h^−1^ at the end. **b** Microreactors dosage is twice as much as **a** flow rate from 0.8 to 0.2 mL h^−1^. **c** Microreactors dosage is three times as much as **a** flow rate from 0.5–0.2 mL h^−1^. **d** Microreactors dosage is four times as much as (**a**), the flow rate from 0.8 to 0.2 mL h^−1^. **e** Flow rate from 1.0 to 0.5 mL h^−1^. **f** Flow rate from 0.8 to 0.3 mL h^−1^. **B** Cascade reaction involving H-beta zeolite and CALB. **g** Cascade reaction for the synthesis of chiral 1-phenylethanol acetate. **h** Conversion and e.e. with time for the batch reaction using H-beta zeolite and CALB as catalysts. **i** Conversion and e.e. with time for the batch reaction using H-beta zeolite and CALB/SCs. **j** Conversion and e.e. with time for the flow reaction with H-beta zeolite and CALB/SCs co-localized in the biomimetic microreactor. **k** Specific activity of CALB in the batch or flow systems. The specific activity of CALB was calculated according to the conversion within the first 2 h for the batch reactions and after the conversion leveled off for the flow reactions. Reaction conditions are provided in Supplementary Information.

Various aldehydes were further examined, including furfuraldehyde, 4-chlorobenzaldehyde, hexaldehyde, butyraldehyde, 4-nitrobenzaldehyde as well as benzaldehyde (Fig. 6A). For all the investigated substrates, after a period of time, the conversion increased to more than 70% and then leveled off reaching a steady state. For furfuraldehyde and 4-chlorobenzaldehyde as substrates (Fig. 6A, a, b), as high as 80–85% conversions were obtained and their e.e. of chiral products were > 99%. After a continuous operation lasting as long as 120 h, their overall conversions and e.e. showed no significant decrease. For less reactive substrates such as hexaldehyde and butyraldehyde (Fig. 6A, c, d), e.e. of chiral products were still >99% over a period of 120 h and the conversions were always maintained between 72 and 81%. For a substrate containing a strong electron-withdrawing substituent, for example, 4-nitrobenzaldehyde, >98% conversion was achieved even over 120 h (Fig. 6A, e). To further examine the durability of the biomimetic microreactor, the running time was prolonged to 240 h (Fig. 6A, f). Throughout such a long period of time, *ca*. 90–92% conversion of benzaldehyde and 99% e.e. of the chiral product was always maintained at a flow rate of 0.3–0.8 mL h^−1^ (the slight decrease in CE may be caused by the loss of 4-dimethylaminopyridine (co-catalyst)). Inductively coupled plasma mass spectrometry (MS) showed that more than 95% of the Ti element in the flow reaction was retained in the IL droplets after 48 h of flow reaction. The retention of CALB was also determined to be 95% according to S content measurement. The reason for the high stability can be ascribed to the confinement effect of the Pickering emulsion droplets, in which the catalytically active SCs can be well entrapped owing to the physical obstruction at droplet interfaces, as discussed above.

To explore the generality of our method, we next applied it to another chemo-enzymatic cascade reaction, combining H-beta zeolite-catalyzed racemization of 1-phenylethanol and CALB-promoted dynamic kinetic resolution of racemic 1-phenylethanol (Fig. 6B, g)^49,50^. Cascading these two reactions in one pot in principle may improve the theoretical yield of the chiral ester from 50 to 100%. First, in the batch cascade system, H-beta zeolite and CALB were directly mixed. The conversion of 1-phenylethanol was only 8% after 12 h (Fig. 6B, h). The low conversion was caused by the incompatibility between the zeolite and enzyme. When H-beta zeolite and CALB/SCs were used in batch reactions, 35% conversion and 99% e.e. was obtained after only 2 h (Fig. 6B, i). When utilizing the biomimetic microreactor-based continuous flow system, the conversion of 1-phenylethanol at steady-state rapidly rose up to 97% and the e.e. was always 99% (Fig. 6B, j). The specific activity of CALB was calculated to be 0.11 U mg^−1^, which is 2.8 times higher than that of H-beta zeolite and CALB/SCs in the batch reaction system, and 16.4 times higher than that of direct mixing of H-beta zeolite and CALB (Fig. 6B, k). Notably, after continuous running for 85 h, the conversions and enantioselectivities showed no apparent decrease. These results again demonstrate the high flexibility of the proposed biomimetic microreactor in processing continuous flow chemo-enzymatic cascade reactions, as well as further underlining that our biomimetic microreactor-based flow system can also significantly improve the CE.

## Discussion

In summary, we have successfully developed a facile method for practical cascade catalysis based on Pickering emulsions and continuous flow. The key to this success is the co-localization of two catalytically active SCs within the droplets via emulsification yielding biomimetic microreactors. This co-localization method not only allows to rationally tune the amount and ratio of SCs within the microreactor but also enables enclosure of the desired liquid medium that is proven capable of sequestering reactants into the microreactor. In two independent chemo-enzymatic cascade reactions involving Ti(Salen)-lipase CALB cascade and H-beta zeolite-CALB cascade, the biomimetic microreactors were demonstrated to work very efficiently for continuous flow synthesis of chiral O-acylated cyanohydrins and chiral 1-phenylethanol acetate, highlighted by maintaining 99% e.e. over 80–240 h. Impressively, the reaction efficiency of the biomimetic microreactor-based continuous flow system was improved 5–420 times relative to a typical batch reaction. The compartmentalization and enriching reactant properties of the microreactor under continuous flow conditions were found to be crucial to the high CE. Moreover, we have successfully established a theoretical model for cascade reactions within the microreactor, making it possible to rationally design the microreactor and predict its reaction kinetics. We anticipate that our methodology and the insights into cascade reactions in biomimetic microreactors will significantly expedite practical cascade catalysis.

## Methods

### General procedures for the preparation of biomimetic microreactors

Silica emulsifier and catalytically active SCs were simultaneously added into a biphasic mixture of [BMIM]PF_6_, PEG-300, water, and *n*-octane (at a volume ratio of 0.7: 1: 0.3: 1). The resultant suspension was emulsified through vigorous shearing (8000 rpm for 2 min) with a homogenizer, yielding Pickering emulsion droplet-based biomimetic microreactors.

### Chemo-enzymatic cascade synthesis of chiral O-acylated cyanohydrins in biomimetic microreactor-based flow systems

A mixture of 2.1 mL [BMIM]PF_6_, 3 mL PEG-300 and 0.9 mL PBS (pH = 7.4, 0.05 M), 0.2 g Ti/SCs, 0.1 g CALB/SCs, 3 mL *n*-octane and 0.18 g emulsifier was stirred at 8000 rpm for 2 min, yielding biomimetic microreactors. The resultant biomimetic microreactors were filled in a glass column reactor (inner diameter is 2 cm) whose bottom is a sand filter (4.5–9 µm in pore diameter). A solution of aldehydes (0.05 M) and acetyl cyanide (0.2 M) in *n*-octane as mobile phase was pumped through the inlet of the column reactor at a given flow rate and was allowed to pass through the column reactor whose temperature was kept at 25 °C. The outflow from the column reactor was sampled for gas chromatography (GC) (equipped with a chiral column) analysis at intervals, and the product was further confirmed with GC–MS.

## Supplementary information

Supplementary Information

## Data Availability

The data for Figs. 2–6 generated in this study are provided in the Supplementary Information/Source Data file. Source data are provided with this paper.

## Source data

Source data
