# Supplementary material for: Pickering emulsion droplet-based biomimetic microreactors for continuous flow cascade reactions
Source: Nat Commun. 2022 Jan 25;13:475. doi: 10.1038/s41467-022-28100-6 (PMC8789915; doi:10.1038/s41467-022-28100-6)
Supplement: Supplementary file 1 — Supplementary Information [file 41467_2022_28100_MOESM1_ESM.pdf]

Supplementary Information for

Pickering emulsion droplet-based biomimetic microreactors for continuous flow cascade reactions

Ming Zhang[1], Rammile Ettelaie[2], Lianlian Dong[1], Xiaolong Li[1], Ting Li[1], Xiaoming Zhang[1], Bernard P. Binks[3], and Hengquan Yang[1,4] *

[1]*School of Chemistry and Chemical Engineering, Shanxi University, Taiyuan 030006, China*

[2]*Food Colloids Group, School of Food Science and Nutrition, University of Leeds, Leeds LS2 9JT, U.K.*

[3]*Department of Chemistry, University of Hull, Hull. HU6 7RX. U.K.*

[4]*Key Laboratory of Chemical Biology and Molecular Engineering of Ministry of Education, Institute of Molecular Science, Shanxi University, Taiyuan 030006, China*

*To whom correspondence should be addressed: hqyang@sxu.edu.cn

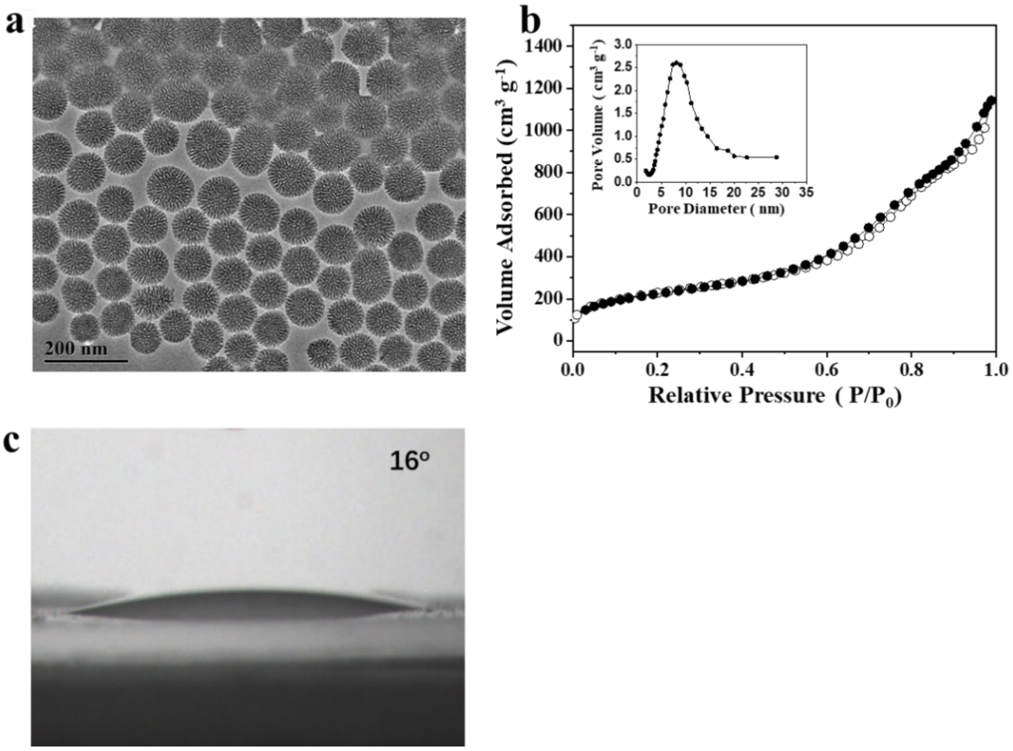

Supplementary Figure 1. Characterization of mesoporous silica nanospheres (MSNs). **a**, TEM image, scale bar = 200 nm. **b**, N_2 adsorption–desorption isotherms and pore size distribution (inset). **c**, Appearance of a water droplet in air on a disk of compressed MSNs and the corresponding contact angle.

Notes：The average particle diameter of MSNs is *ca*. 80−90 nm, as measured by TEM (Supplementary Fig. 1a). The BET specific surface area was measured to be 832 m^2 g^{-1} and the average pore size is *ca*. 8.0 nm, calculated from the BJH method (Supplementary Fig. 1b, inset).

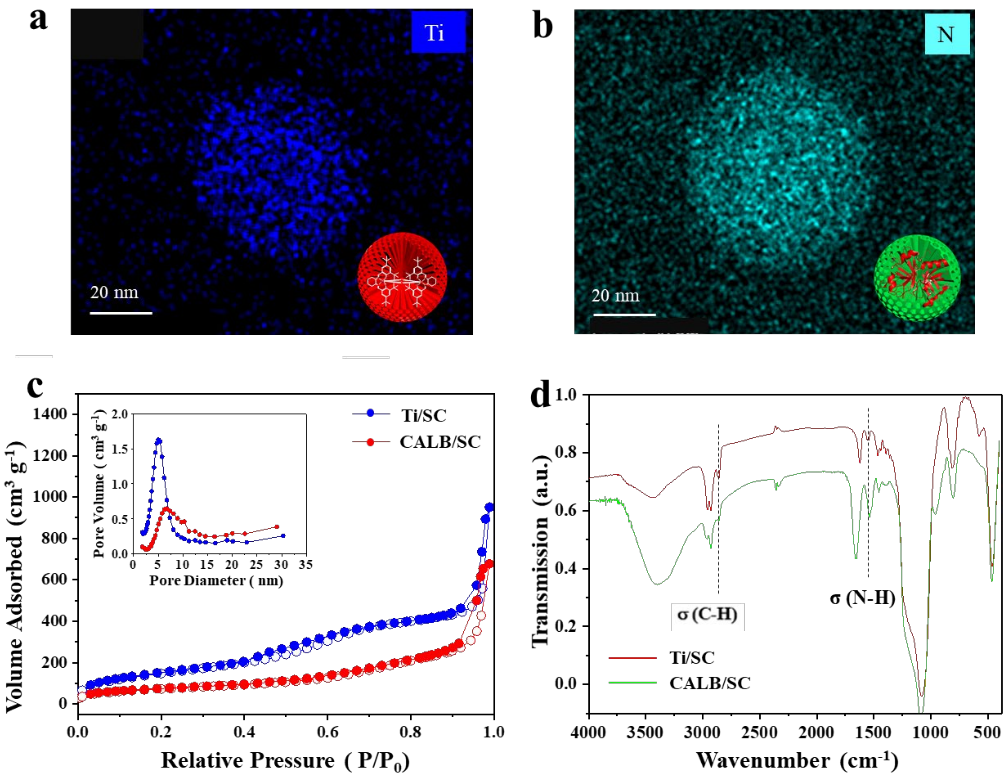

Supplementary Figure 2. Characterization of Ti/SCs and CALB/SCs. **a**, EDS mapping of Ti/SCs for Ti. **b**, EDS mapping of CALB/SCs for N. **c**, N_2 adsorption–desorption isotherms and the corresponding pore size distributions (inset). The specific surface areas are 548 and 267 m^2 g^{-1} respectively for Ti/SCs and CALB/SCs, while the pore sizes are 5.0 nm and 6.7 nm. **d**, FTIR spectra of Ti/SCs and CALB/SCs.

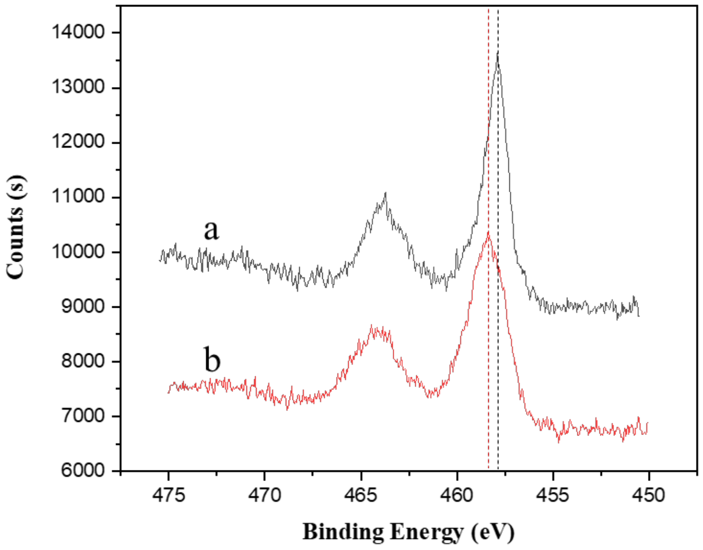

Supplementary Figure 3. XPS spectra of samples. a, Ti(Salen); **b,** Ti/SCs.

Notes: From the above Ti 2*p* spectra, it can be clearly observed that the binding energy of Ti $2p_{3/2}$ peak for Ti/SCs has a 0.5 eV shift compared to that of Ti(Salen), indicating that there exist interactions between MSNs and Ti(Salen). Moreover, a filtration test was also performed to further examine the Ti(Salen) leaching. After 15 cycles at a stirring system (15 min each time), only 14% of Ti(Salen) was detected in the residual filtrate. Furthermore, we found that the filtrate of the reaction system is almost inactive towards the asymmetric addition of acetyl cyanide to aldehydes.

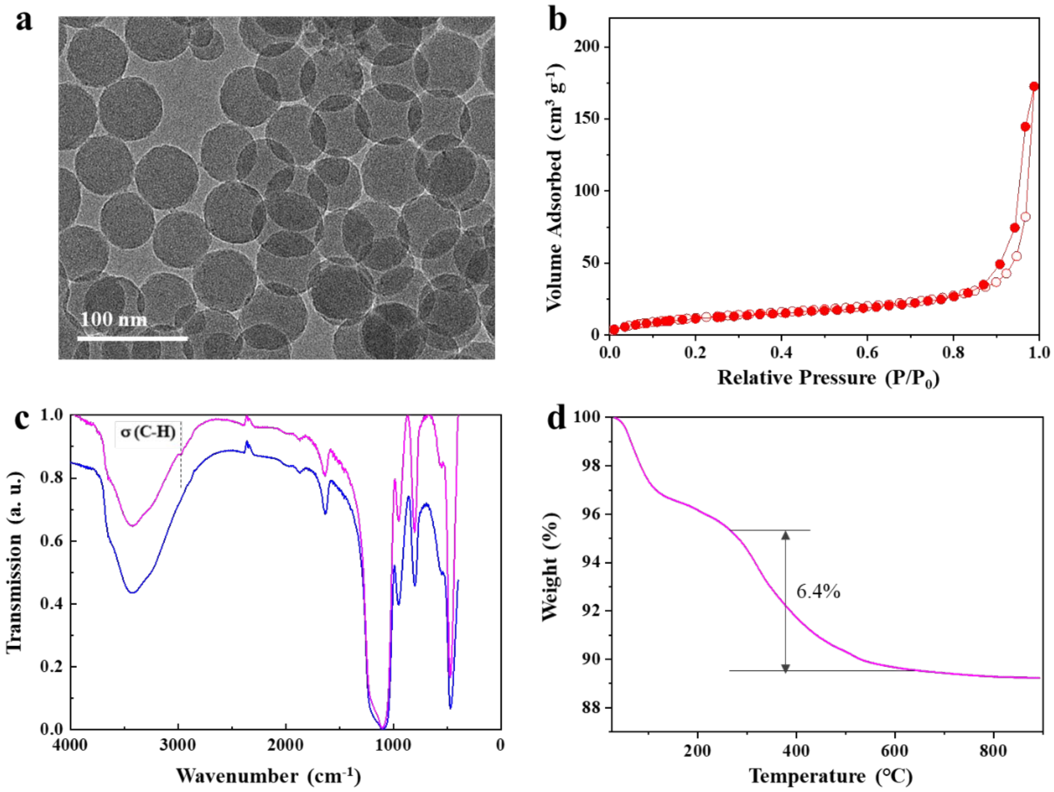

Supplementary Figure 4. Characterization of silica emulsifier. **a**, TEM image, scale bar = 100 nm. **b**, N_2 adsorption-desorption isotherms. **c**, FTIR spectra of silica particles before (blue line) and after (pink line) hydrophobic modification. **d**, TGA graph of silica emulsifier.

Notes： The BET specific surface area is 47 m^2 g^{-1} indicating the particles are non-porous (Supplementary Fig. 3b).

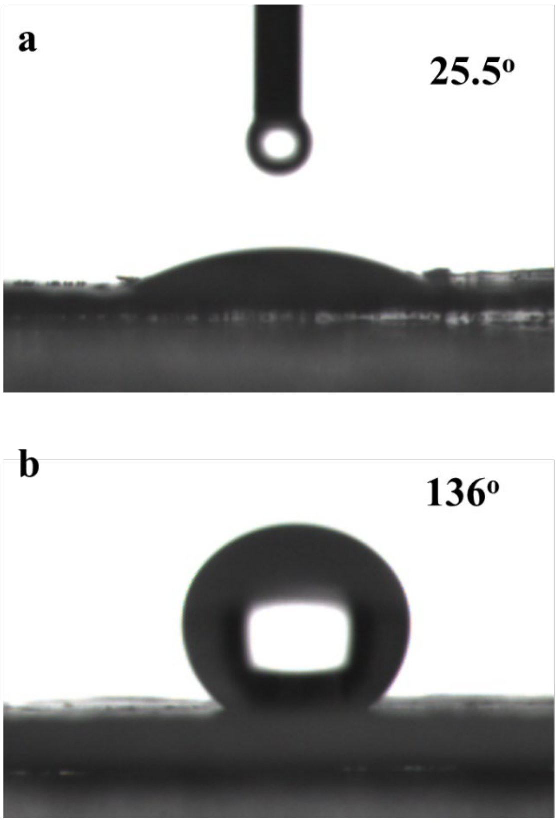

Supplementary Figure 5. Appearance of a water droplet in air on a disk of compressed silica particle emulsifier and the corresponding contact angle. Unmodified silica (**a**) and hydrophobically modified silica (**b**).

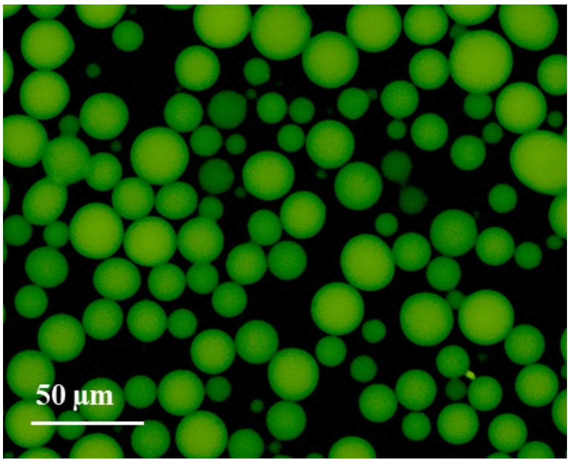

Supplementary Figure 6. Fluorescence confocal microscopy image for the IL-in-oil droplet microreactors with the IL dyed by FITC-I. Scale bar = 50 µm. The Pickering emulsion consists of 0.7 mL [BMIM]PF_6, 1 mL PEG-300, 0.3 mL PBS (pH = 7.4, 0.05 M), FITC-I (2 µM, with respect to the volume of IL phase), 1 mL *n*-octane and 0.03 g silica emulsifier.

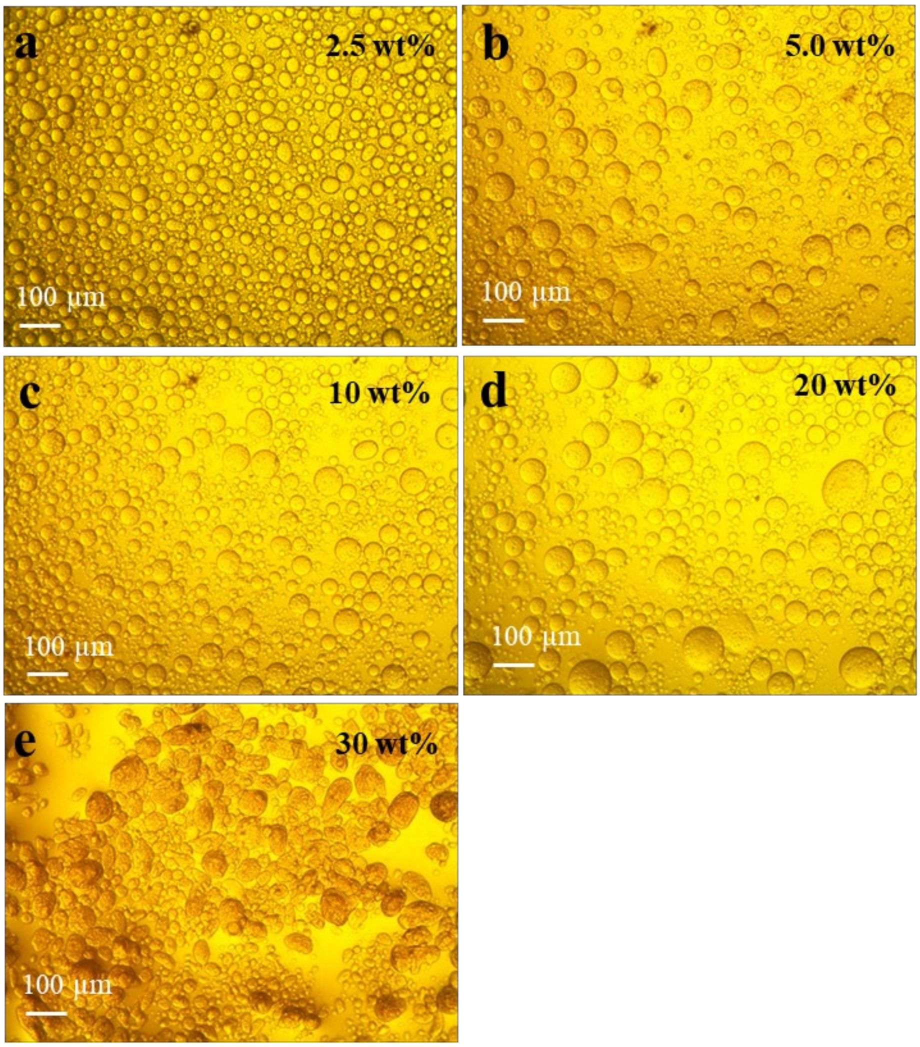

Supplementary Figure 7. Optical micrographs of IL-in-oil droplet microreactors containing different amounts of MSNs. The Pickering emulsions consist of 0.7 mL [BMIM]PF_6, 1 mL PEG-300, 0.3 mL phosphate buffer solution (PBS: 50 mM Na_2HPO_4-50 mM NaH_2PO_4, pH = 7.4), 1 mL *n*-octane, 0.03 g silica emulsifier, and different concentrations of MSNs (with respect to the weight of IL): **a**, 2.5 wt%; **b**, 5.0 wt%; **c**, 10 wt%; **d**, 20 wt%; **e**, 30 wt%.

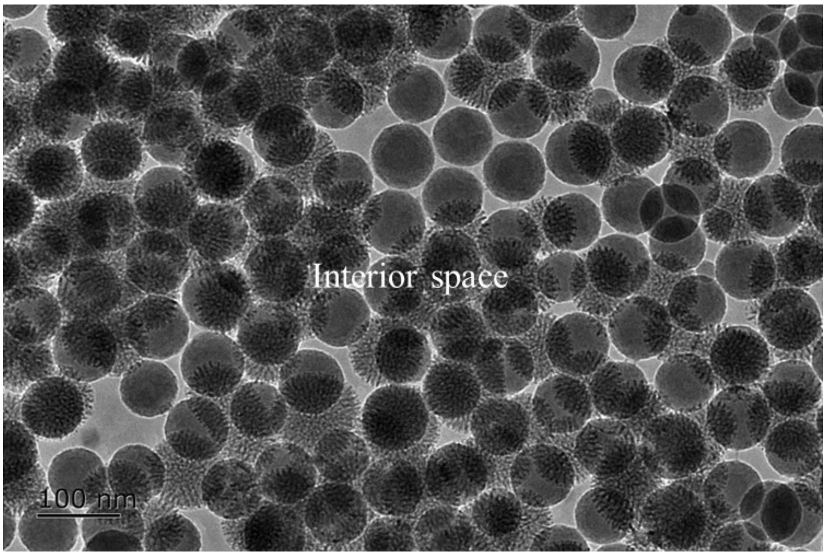

Supplementary Figure 8. Magnified cryo-TEM image of a frozen SCs-containing water droplet in oil. This image shows the distribution of SCs within the interior of the microreactors.

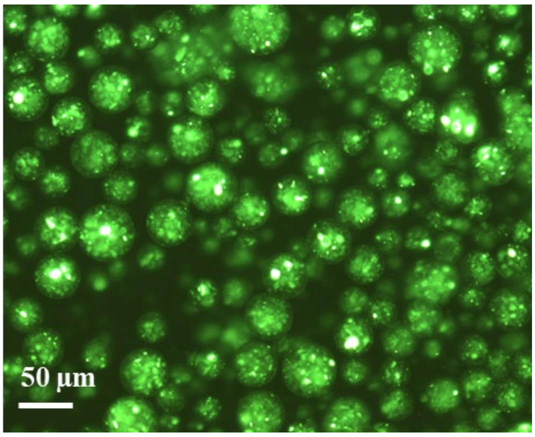

Supplementary Figure 9. Confocal laser scanning microscopy image of the MSNs (dyed with FITC-I)-containing IL-in-oil droplet microreactors after violent shaking. Scale bar = 50 μm. The Pickering emulsion consists of 0.7 mL [BMIM]PF_6, 1 mL PEG-300, 0.3 mL PBS (pH = 7.4, 0.05 M), 1 mL *n*-octane, 15 mg FITC-I-labelled MSNs, 0.03 g silica emulsifier.

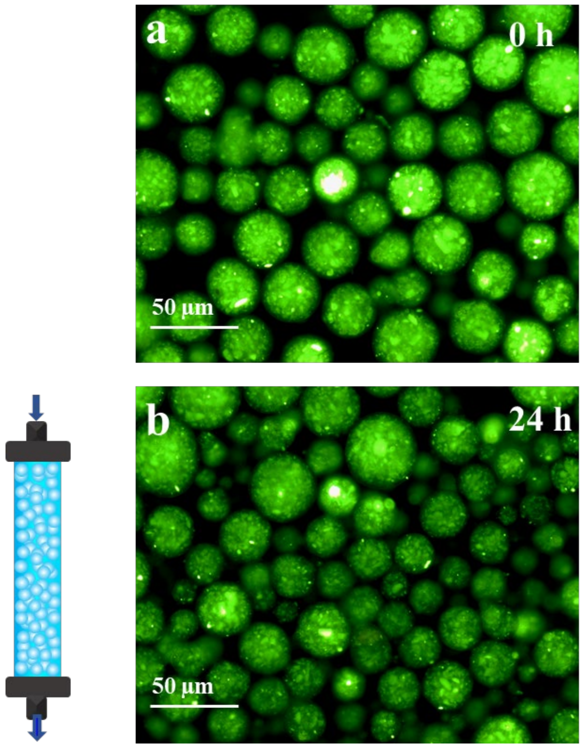

Supplementary Figure 10. Confocal laser scanning microscopy images of the MSNs (dyed with FITC-I)-containing IL-in-oil droplet microreactors before and after continuous flow. a, before continuous flow; **b**, after 24 h of continuous flow. The Pickering emulsion consists of 0.7 mL [BMIM]PF_6, 1 mL PEG-300, 0.3 mL PBS (pH = 7.4, 0.05 M), 1 mL *n*-octane, 15 mg FITC-I-labelled MSNs, 0.03 g silica emulsifier.

Notes: To check whether the flow of oil leads to the leakage of MSNs, we employed fluorescence microscopy to observe the MSNs positions before and after 24 h of flow. As shown in Supplementary Fig. 7, after flowing for 24 h the fluorescently-labelled MSNs were still located within droplets. No fluorescence signals were observed outside the droplets, demonstrating a high level of encapsulation efficiency and a good entrapment ability of the IL droplets towards the SCs.

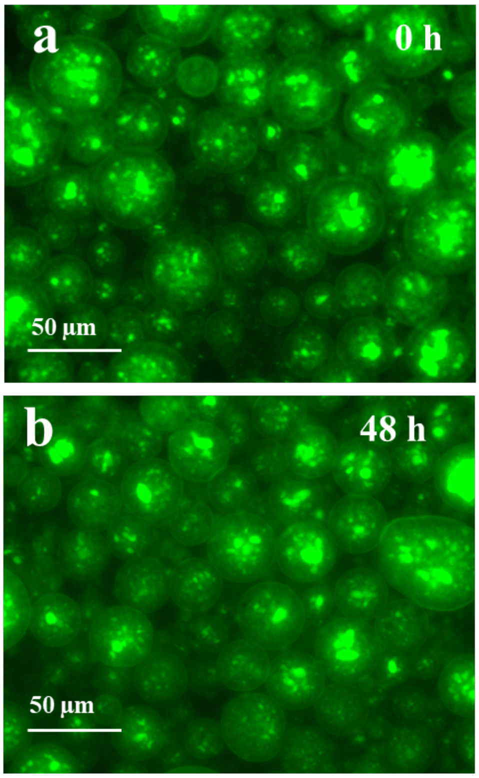

Supplementary Figure 11. Confocal laser scanning microscopy images of the MSNs (dyed with FITC-I)-containing IL-in-oil droplet microreactors. a, before standing for 48 h; **b**, after standing for 48 h. The Pickering emulsion consists of 0.7 mL [BMIM]PF_6, 1 mL PEG-300, 0.3 mL PBS (pH = 7.4, 0.05 M), 1 mL *n*-octane, 15 mg FITC-I-labelled MSNs, 0.03 g silica emulsifier.

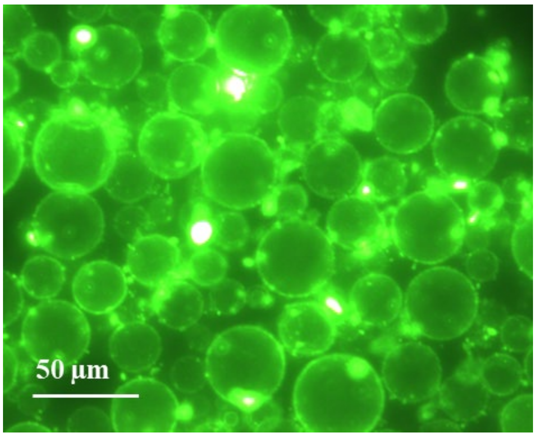

Supplementary Figure 12. Confocal laser scanning microscopy image of the MSNs (dyed with FITC-I)-containing water-in-oil droplet microreactors with water as dispersed phase. The Pickering emulsion consists of 2 mL PBS (pH = 7.4, 0.05 M), 1 mL *n*-octane, 15 mg FITC-I-labelled MSNs, 0.03 g silica emulsifier.

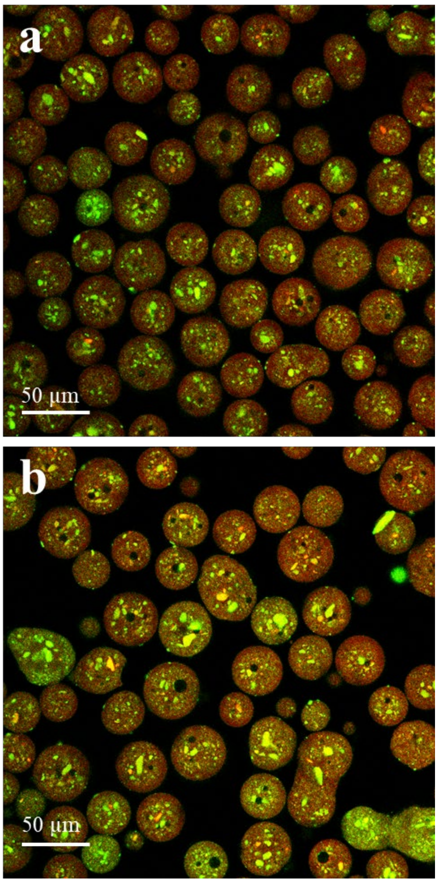

Supplementary Figure 13. Confocal laser scanning microscopy images of the two SCs (Rhodamine B-labelled Ti/SCs and FITC-I-labelled CALB/SCs)-containing IL-in-oil droplet microreactors before and after continuous flow reaction. **a**, before continuous flow reaction; **b**, after 48 h of continuous flow reaction. The Pickering emulsion consists of 0.7 mL $[BMIM]PF_6$, 1 mL PEG-300, 0.3 mL PBS (pH = 7.4, 0.05 M), 1 mL *n*-octane, 10 mg Rhodamine B-labelled Ti/SCs, 5 mg FITC-I-labelled CALB/SCs and 0.03 g silica emulsifier.

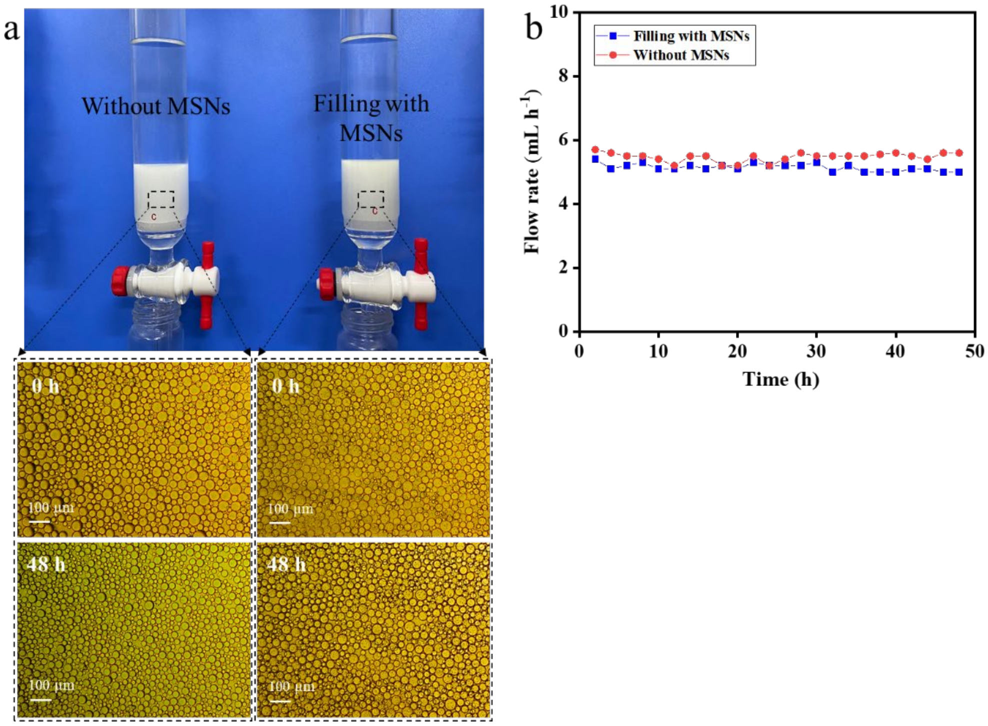

Supplementary Figure 14. Comparison of flow behavior between the microreactors hosting solid particles and the microreactors without the solid particles. a, Appearance of the two microreactors in the column and their optical micrographs before and after 48 h of continuous flow. **b**, Flow rate of the oil with time for these two systems.

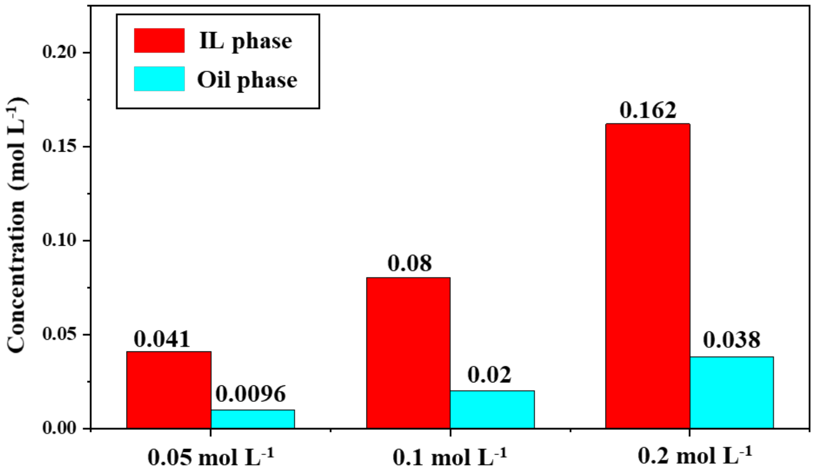

Supplementary Figure 15. Partitioning of benzaldehyde between IL phase and oil at different benzaldehyde concentrations. A mixture of 1 mL oil solution containing a given concentration of benzaldehyde (0.05, 0.1 or 0.2 M) and 1 mL of IL phase containing [BMIM]PF_6, PEG-300 and PBS (Volume ratio is 0.7:1:0.3) was stirred for 12 h at room temperature to reach equilibrium. After phase separation, the concentration of benzaldehyde in the oil phase was determined by GC. The concentration of benzaldehyde in the IL phase is calculated from the difference between the initial concentration in the oil and the determined concentration after equilibrium. This experiment was repeated three times to obtain an average value. The partition coefficient α of benzaldehyde between the IL phase and the oil phase is the ratio of the benzaldehyde concentration in the ionic liquid to that in the oil at equilibrium. By using this method, the partition coefficients of benzaldehyde, acetyl cyanide and O-acylated cyanohydrin were determined to be 4.2, 28 and 26, respectively (average values from different initial concentrations in oil).

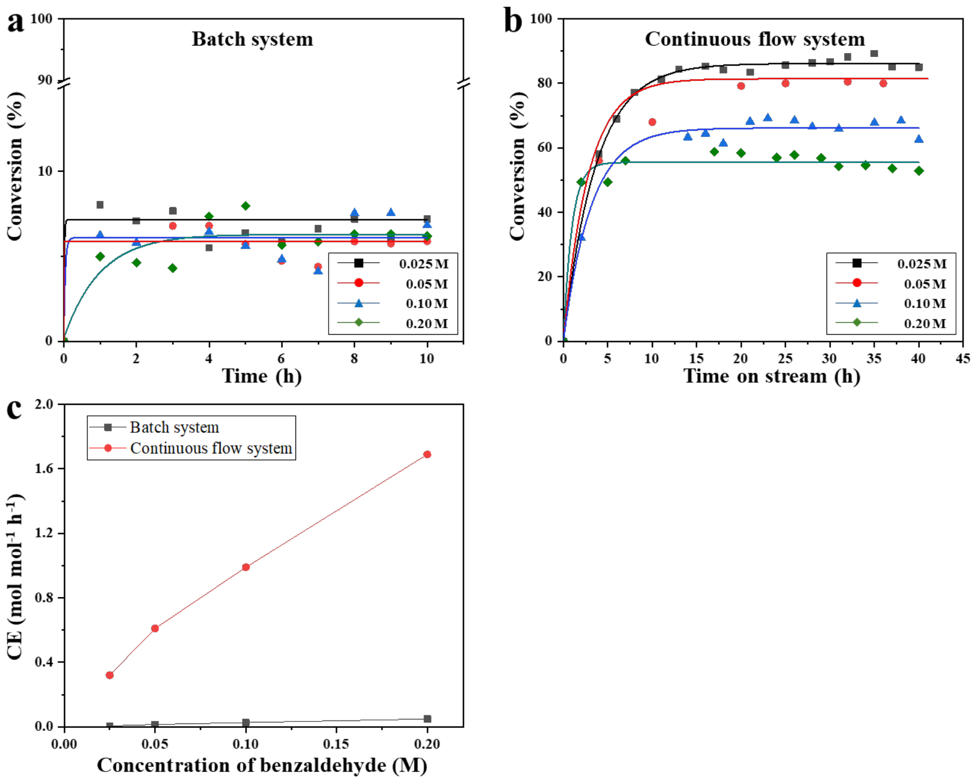

Supplementary Figure 16. Ti(Salen)-CALB cascade synthesis of chiral O-acylated cyanohydrin in batch and continuous flow systems with different concentrations of benzaldehyde. a, Conversion as a function of time in the batch system. **b,** Conversion as a function of time in the continuous flow system. **c**, CEs at different concentrations of benzaldehyde in the batch system were calculated within first 10 h, and CEs in the continuous flow system at different concentrations of benzaldehyde at steady-state. Reaction conditions: the system consists of 2.1 mL [BMIM]PF_6, 3 mL PEG-300, 0.9 mL PBS (pH = 7.4, 0.05 M), 0.2 g Ti/SCs, 0.1 g CALB/SCs, 3 mL of *n*-octane, 0.18 g emulsifier. The concentration of benzaldehyde in *n*-octane was varied from 0.025 to 0.2 M (fixing the molar ratio of benzaldehyde and acetyl cyanide at 1 : 4), 900 rpm, 25 °C. For the flow system, flow rate = 1 mL h^{-1}.

Notes: With increasing the reactant concentration, we found that the catalysis efficiency in the flow system increased more pronouncedly than the batch reaction.

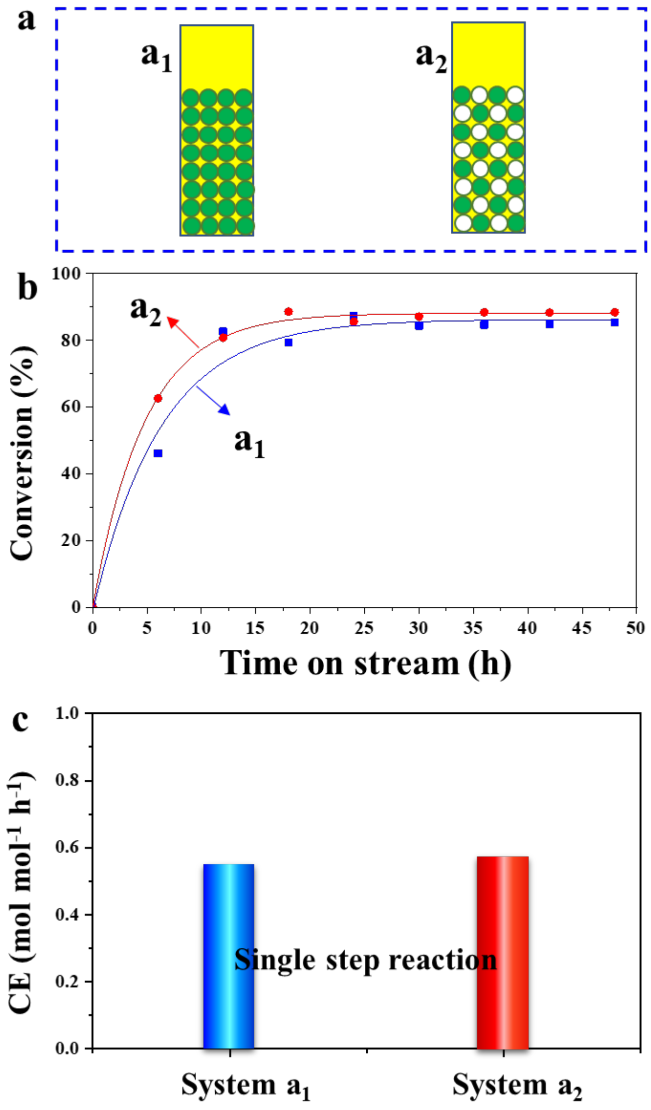

Supplementary Figure 17. Ti(Salen)-catalyzed synthesis of chiral O-acylated cyanohydrin in different systems. **a**, Schematic illustration of two reaction systems: Ti/SCs was uniformly distributed in all of microreactors (**a**$_1$) or confined in half of microreactors (**a**$_2$). **b**, Conversion as a function of time in the continuous flow systems. **c**, CE of system a$_1$ and system a$_2$ calculated after the conversion leveled off. The biomimetic microreactor-based flow system consists of 2.1 mL [BMIM]PF$_6$, 3 mL PEG-300, 0.9 mL PBS (pH = 7.4, 0.05 M), 0.2 g Ti/SCs, 3 mL of *n*-octane, 0.18 g emulsifier, solution of benzaldehyde (0.05 M) and acetyl cyanide (0. 2 M) in *n*-octane as mobile phase, 25 °C, flow rate = 2 mL h^{-1}. The total dosage of Ti/SCs is fixed for both reaction systems.

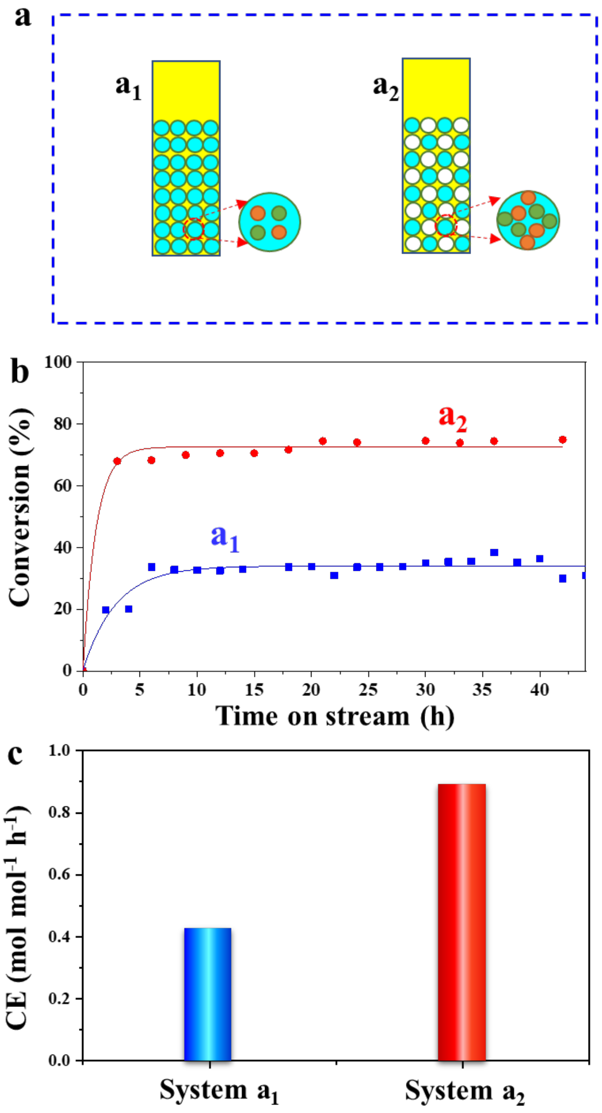

Supplementary Figure 18. Ti(Salen)-CALB cascade synthesis of chiral O-acylated cyanohydrin in different systems. **a**, Schematic illustration of two reaction systems: two catalytically active SCs were uniformly distributed in all of microreactors (**a**$_1$) or only confined in half of microreactors (**a**$_2$). **b**, Conversion as a function of time in the continuous flow chemo-enzymatic cascade reaction systems. **c**, CEs of system a$_1$ and system a$_2$, calculated after the conversion leveled off. Reaction conditions: the biomimetic microreactor-based flow system consists of 2.1 mL [BMIM]PF$_6$, 3 mL PEG-300, 0.9 mL PBS (pH = 7.4, 0.05 M), 0.2 g Ti/SCs, 0.1 g CALB/SCs, 3 mL *n*-octane, 0.18 g emulsifier, solution of benzaldehyde (0.05 M) and acetyl cyanide (0. 2 M) in *n*-octane as mobile phase, 25 °C, flow rate = 2 mL h^{-1}. The total dosage and the ratio of the two SCs are fixed for both of reaction systems.

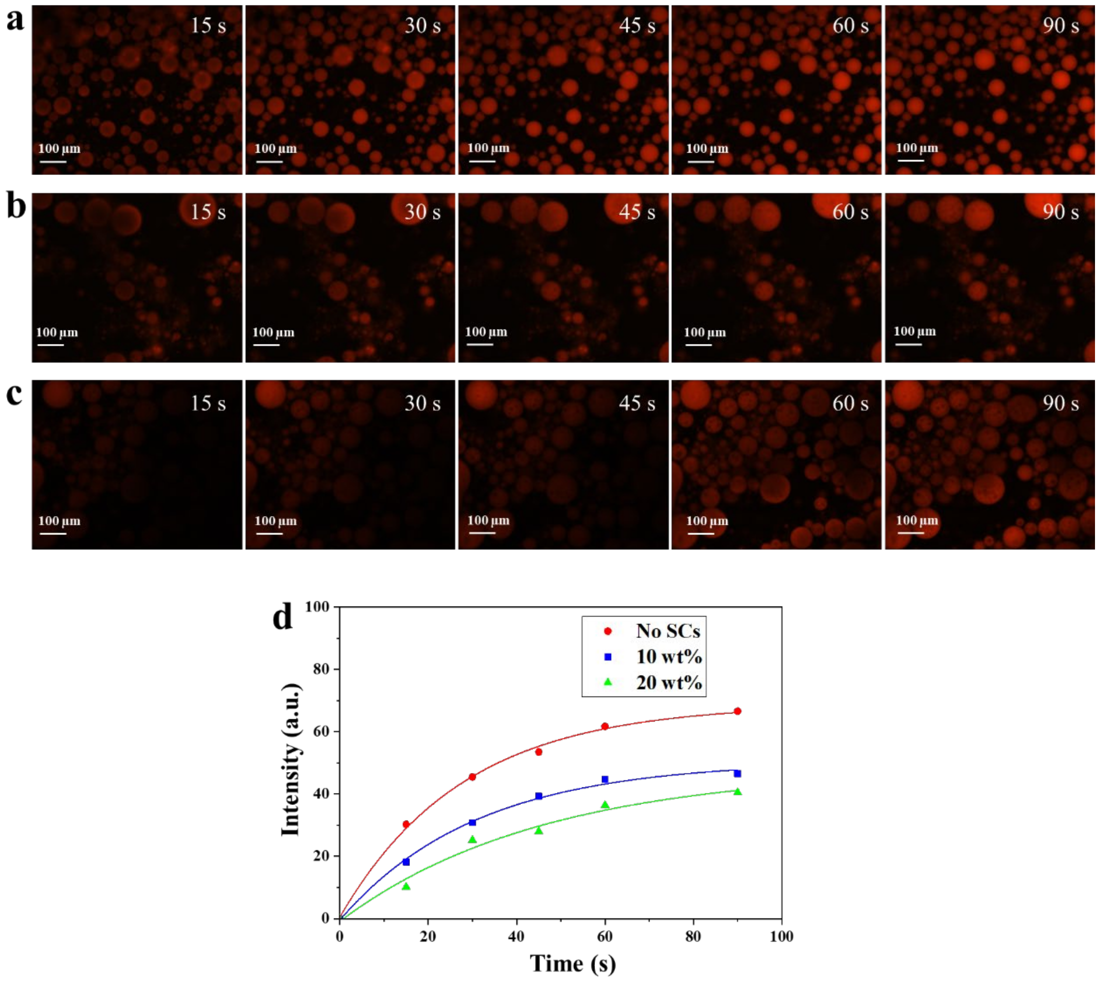

Supplementary Figure 19. Fluorescence microscopy images with time for the transport of probe molecules (5 μM Nile Red) into droplet microreactors. No SCs (**a**) or 10 wt% (**b**) and 20 wt% (**c**) of SCs were encapsulated inside the microreactors, respectively. **d**, Fluorescence intensity as a function of time for the transport of Nile Red into biomimetic microreactor. The Pickering emulsion consists of 0.7 mL [BMIM]PF_6, 1 mL PEG-300, 0.3 mL PBS (pH = 7.4, 0.05 M), 1 mL *n*-octane, 0.06 g emulsifier and different concentrations of SCs.

Notes: The fluorescent reagent Nile Red initially present in the continuous oil phase was observed to spontaneously enter the IL droplets as shown by the time-dependent fluorescence microscopy images of the IL droplets (Supplementary Fig. 11a-c) and the time-course fluorescence intensity (Supplementary Fig. 11d). The results show that an increase in the SCs concentration can lead to a lower molecular diffusion rate.

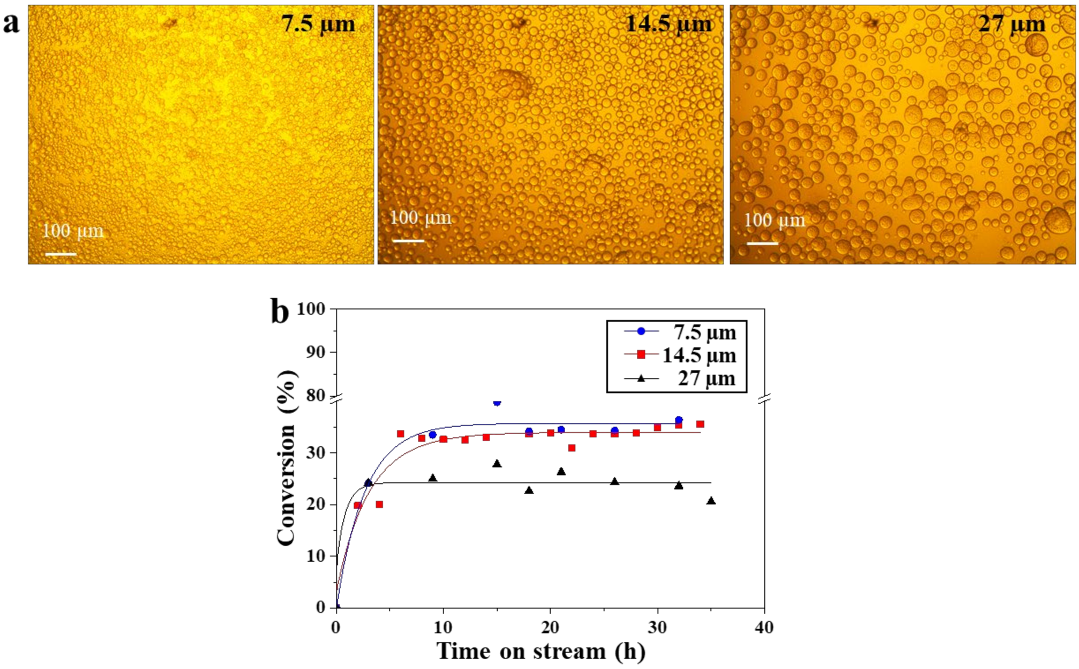

Supplementary Figure 20. Ti(Salen)-CALB cascade synthesis of chiral O-acylated cyanohydrin in the continuous flow system with different microreactor sizes. **a**, Optical micrographs of droplet microreactors with different diameters. Scale bar = 100 μm. **b**, Conversion as a function of time in the biomimetic microreactor with different sizes in continuous flow systems. The biomimetic microreactor-based flow system consists of 2.1 mL [BMIM]PF_6, 3 mL PEG-300, 0.9 mL PBS (pH = 7.4, 0.05 M), 0.2 g Ti/SCs, 0.1 g CALB/SCs, 3 mL of *n*-octane, and a given amount of solid emulsifier.

Notes: The fine-tuning of microreactor droplet size was achieved by varying the amount of emulsifier. As the amount of emulsifier was increased from 1 to 3 and 6 wt%, the average microreactor droplet radius decreased from *ca*. 27 to 14.5 to 7.5 μm (Supplementary Fig. 14a).

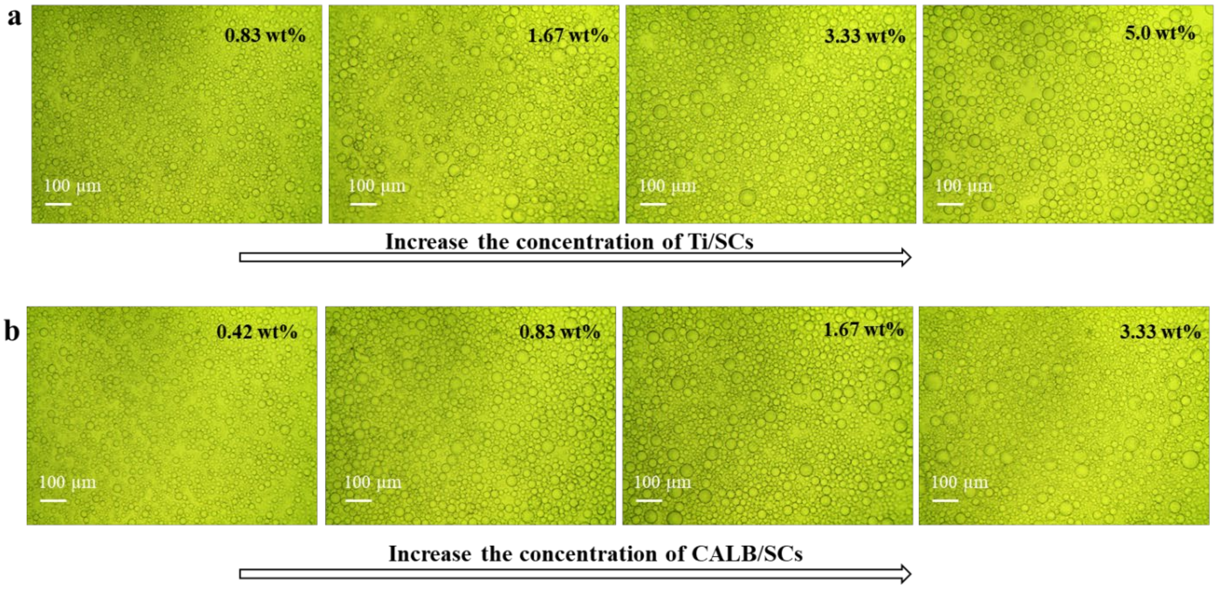

Supplementary Figure 21. Optical micrographs of droplet microreactors containing different ratios of Ti/SCs and CALB/SCs. **a**, Concentration of Ti/SCs was varied from 0.83 to 1.67, 3.33, 5.0 wt% while the concentration of CALB/SCs constant at 1.67 wt%. **b**, Concentration of CALB/SCs was varied from 0.42 to 0.83, 1.67, 3.33 wt% while the concentration of Ti/SCs kept constant at 3.33 wt%. The Pickering emulsions consist of 0.7 mL [BMIM]PF_6, 1 mL PEG-300, 0.7 mL PBS (pH = 7.4, 0.05 M), 1 mL *n*-octane, 0.03 g silica emulsifier and different concentrations of SCs.

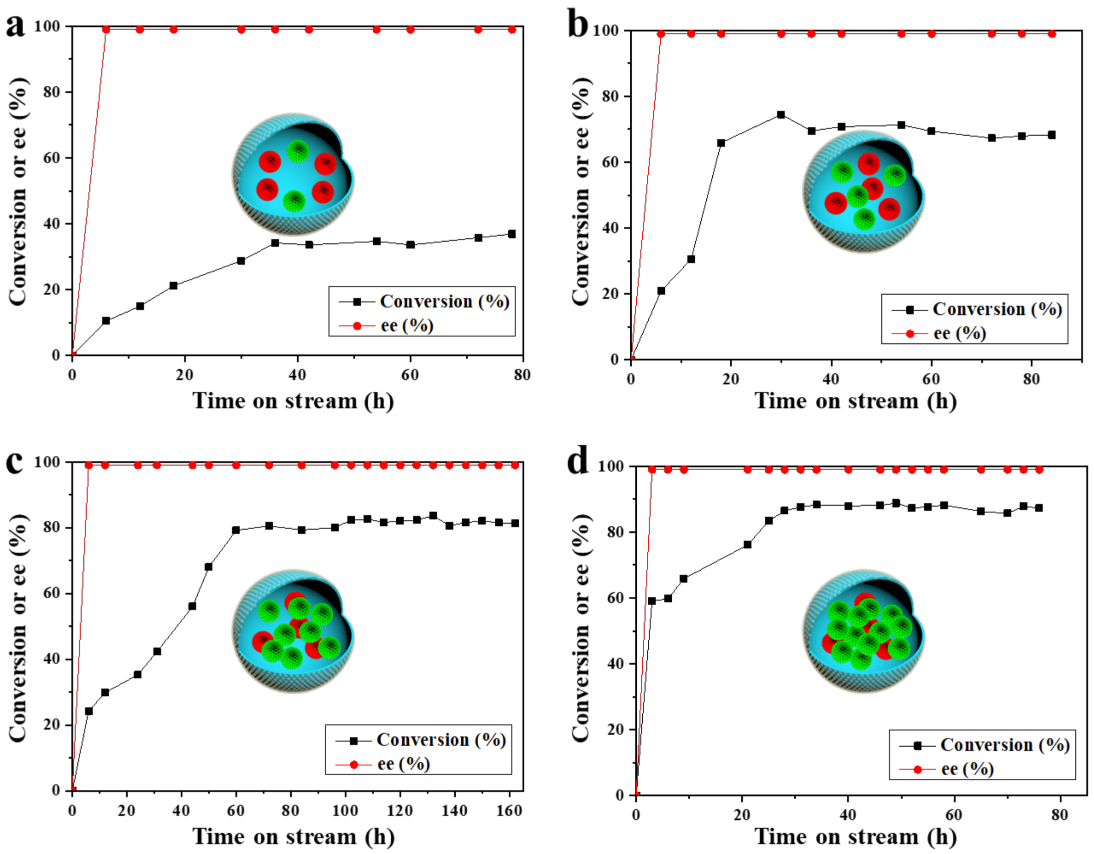

Supplementary Figure 22. Ti(Salen)-CALB cascade synthesis of chiral O-acylated cyanohydrin in the continuous flow systems at different concentrations of Ti/SCs. The biomimetic microreactor-based flow system consists of 2.1 mL [BMIM]PF_6, 3 mL PEG-300, 0.9 mL PBS (pH = 7.4, 0.05 M), a given concentration of Ti/SCs (varied from 0.83 wt% (**a**); 1.67 wt% (**b**); 3.33 wt% (**c**) and 5.0 wt% (**d**)), 1.67 wt% CALB/SCs, 3 mL *n*-octane, 0.18 g emulsifier, solution of benzaldehyde (0.05 M) and acetyl cyanide (0. 2 M) in *n*-octane as mobile phase, 25 °C, flow rate = 1 mL h^{-1}.

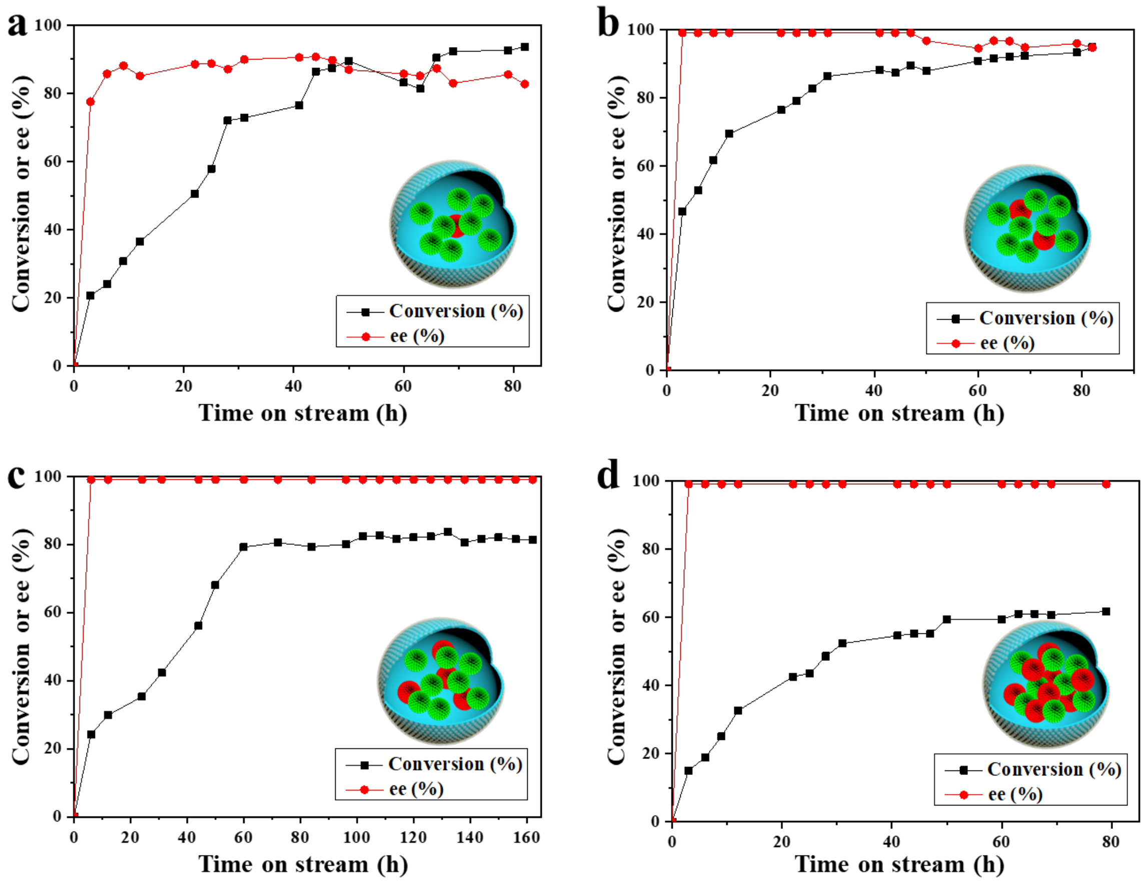

Supplementary Figure 23. Ti(Salen)-CALB cascade synthesis of chiral O-acylated cyanohydrin in the continuous flow system at different concentrations of CALB/SCs. The biomimetic microreactor-based flow system consists of 2.1 mL [BMIM]PF_6, 3 mL PEG-300, 0.9 mL PBS (pH = 7.4, 0.05 M), 3.33 wt% of Ti/SCs, a given concentration of CALB/SCs (varied from 0.42 wt% (**a**); 0.83 wt% (**b**); 1.67 wt% (**c**) and 3.33 wt% (**d**)), 3 mL of *n*-octane, 0.18 g emulsifier, solution of benzaldehyde (0.05 M) and acetyl cyanide (0. 2 M) in *n*-octane as mobile phase, 25 °C, flow rate = 1 mL h^{-1}.

Supplementary Methods

1. Chemicals

Dimethyldichlorosilane, *n*-octyltrimethoxysilane, benzaldehyde (98%), furfuraldehyde (99%), 4-chlorobenzaldehyde (98%), 4-nitrobenzaldehyde (97%), *n*-hexylamine (99%), triethylamine (TEA, 99%), rhodamine B (99%), hexadecyltrimethylammonium chloride (CTAC, 97%), titanium isopropoxide (95%), (*S*, *S*)-(+)-N,N'-Bis(3,5-di-tert-butylsalicylidene)-1,2-cyclohexanediamine (98%), 4-dimethylaminopyridine (99%), (*R*, *S*)-1-phenylethyl alcohol (98%), vinyl acetate (99%), and fluorescein isothiocyanate isomer I (FITC-I, CAS No.3326-32-7) were purchased from Aladdin (China). Tetraethyl orthosilicate (TEOS, AR) and *n*-octane (98%) were purchased from Sinopharm Chemical Reagent Co., Ltd, (China). Acetyl cyanide (90%) was obtained from Sigma-Aldrich. H-beta zeolite was purchased from Nankai University Catalyst Co., Ltd, (China). 1-Butyl-3-methylimidazolium hexafluorophosphate ([BMIM]PF_6, 99%) and 1-butyl-3-methylimidazolium bis((trifluoromethyl)sulfonyl)imide ([BMIM]NTf_2, 99%) were purchased from Lanzhou Institute of Chemical Physics, Chinese Academy of Sciences. Native Lipase B from *Candida Antarctica* (CALB) was purchased from Novozymes. Water used in this study was de-ionized water.

2. Characterization

Nitrogen-sorption analysis was performed at −196 °C on a Micromeritics ASAP 2020 analyzer. Before measurement, samples were out gassed at 120 °C under vacuum for 6 h. The specific surface area was calculated from the adsorption branch in the relative pressure range of 0.05−0.15 using the Brunauer-Emmett-Teller (BET) method. Samples for transmission electron microscopy (TEM) observation were prepared by dispersing the sample powder in ethanol using ultrasound and then allowing a drop of the suspension to evaporate on a copper gird covered with a holey carbon film. TEM images were obtained on a JEOL-JEM-2000EX instrument. Cryo-TEM images were obtained using a JEOL-2100F instrument, and samples were kept at −170 °C during the TEM observations. Emulsion droplets were observed using an optical microscope analyzer (XSP-8CA, China) equipped with 10 × magnification lens. FT-IR spectra were collected with a Bruker Tensor II spectrophotometer in the range 400−4000 cm^{-1}. The content of elemental titanium was determined with an Agilent 720ES inductively coupled plasma optical emission spectrometer (ICP-OES). The content of elemental sulfur was determined with an Elementar Vario EL CHNS analyzer. The contact angles of water in air on silica particle disks

were measured using a Krüss DSA100 instrument. Before measurement, the powder sample was compressed into a disk of thickness approximately 1 mm (*ca.* 2 MPa). A drop of water (1 μL) was placed on the sample disk. The appearance of the water droplet was recorded at *ca.* 0.1 s with a digital camera. The value of the contact angle was determined by a photogoniometric method. Gas chromatography (GC) analysis was carried out on an Agilent 7890 analyzer (Agilent-19091G-B213, HP-CHIRAL-20B) with a flame ionization detector. The identification of products by mass spectrometry (MS) was performed on a GC-MS instrument (7890B-5977A, HP-5, Agilent). Confocal laser scanning microscopy images were obtained on a Carl Zeiss LSM880 instrument (Germany). The excitation wavelength of FITC-I is 488 nm (green). The excitation wavelength of Rhodamine B is 554 nm (red).

3. Material synthesis

Preparation of mesoporous silica nanospheres (MSNs). MSNs were prepared according to a previously reported method[1]. 0.36 g TEA was added into 120 mL CTAC aqueous solution (10 wt%). The resultant mixture was gently stirred at 60 °C for 1 h. Then, a solution of TEOS in cyclohexane (20 v/v%, 40 mL) was slowly added to the above suspension, which was maintained at 60 °C in a water bath for 12 h under magnetic stirring. The solid product was collected by centrifugation and washed for several times with ethanol. After calcination in air at 550 °C for 5 h (ramping rate, 2 °C min^{-1}), MSNs were obtained.

Preparation of Ti(Salen)-containing sub-compartments (Ti/SCs). A dichloromethane solution of 0.08 g of Ti(Salen) (synthesized according to a reported method[2]) and 0.03 g of 4-dimethylaminopyridine (as cocatalyst) was added dropwise to a dispersion of MSNs (0.2 g). The resultant mixture was further sonicated for 20 min. After evaporation of dichloromethane, Ti/SCs was produced.

Preparation of CALB-containing sub-compartments (CALB/SCs). Before enzyme immobilization, the MSNs were modified with hydrophobic organosilane. In a typical procedure, 1.0 g of as-synthesized MSNs (dried at 120 ºC for 4 h) was dispersed into 20 mL toluene, followed by adding a mixture of *n*-octyltrimethoxysilane (0.3 mmol) and TEA (as catalyst, 0.3 mmol). After refluxing for 4 h under N_2 atmosphere, the solid was isolated *via* centrifugation, and washed four times with toluene,

yielding *n*-octyl-modified MSNs. Then, 0.5 g modified MSNs was added into 40 mL enzyme solution consisting of 10 mL crude CALB (8.0 mg mL^{-1} of protein) and 30 mL phosphate buffer solution (PBS: 50 mM Na_2HPO_4-50 mM NaH_2PO_4, pH = 7.4). During this process, 10 mL ethanol was also added to increase the dispersion of MSNs in water. After slowly rotating at 35 °C for 12 h, the solid material was isolated by filtration, and washed three times with PBS (pH=7.4, 0.05 M). The resulting material was dispersed into 10 mL of dry acetone, filtered out and dried under vacuum. The final obtained material is denoted as CALB/SCs. The loading of CALB on the solid material was calculated on the basis of the difference in the CALB concentration in the PBS before and after adsorption (Bradford method).

Preparation of silica emulsifier. Silica nanoparticles were prepared through a modified Stöber method[3]. 1.0 g of as-synthesized silica nanospheres was dried at 120 °C for 4 h, and then dispersed into 12 mL toluene with sonication for 20 min. 3 mmol dimethyldichlorosilane and 6 mmol *n*-hexylamine (as catalyst) were then added into this suspension. The resultant suspension was stirred under N_2 atmosphere at 60 °C for 4 h. The solid particles were collected through centrifugation, washed with toluene and dried, yielding the silica emulsifier.

Preparation of fluorescently labelled silica materials. 1.0 g of silica materials (such as MSNs, catalyst-containing MSNs or silica emulsifier) and 0.02 mmol 3-aminopropyltriethoxysilane (APTES) were dispersed into 8 mL toluene. After stirring for 2 h under a N_2 atmosphere at 60 °C, the solid materials were collected through centrifugation, washed four times with toluene and dried, yielding amino-functionalized silica. 0.5 g of the amino-functionalized silica and 5 mg fluorescein isothiocyanate isomer I (FITC-I) were re-dispersed into 50 mL ethanol. The mixture was stirred overnight in the dark at room temperature. After centrifugation, the solid was collected and then washed five times with ethanol, yielding FITC-I-labelled silica. For the preparation of Rhodamine B-labelled silica, samples were prepared by a similar method but with water as the solvent.

4. Chemo-enzymatic cascade reactions

Chemo-enzymatic cascade synthesis of chiral O-acylated cyanohydrins in batch reaction. For the Ti(Salen)-CALB system (system A), 2.1 mL [BMIM]PF_6, 3 mL PEG-300 and 0.9 mL PBS (pH = 7.4,

0.05 M), 0.08 g Ti(Salen), 0.03 g 4-dimethylaminopyridine (co-catalyst), 0.9 mL CALB (8.0 mg mL^{-1} of protein), 3 mL *n*-octane containing aldehydes (0.05 M) and acetyl cyanide (0.2 M) were mixed in a 25 mL Schlenk tube, and stirred at 25 °C with a magnetic bar (10 mm in length, 600 rpm). Aliquots of the solution were taken at intervals and extracted with diethyl ether to monitor the conversions and enantiomeric excess (e.e.) by chiral column equipped GC analysis. As for the Ti/SCs-CALB/SCs system, procedures are similar as above except that the catalyst was replaced with Ti/SCs and CALB/SCs (without compartmentalization) or biomimetic microreactor.

H-beta zeolite-CALB cascade catalytic synthesis of chiral ester in batch reaction. 4 mL [BMIM]NTf$_2$, 0.02 g H-beta zeolite, 0.9 mL CALB (8.0 mg mL^{-1} of protein) or 0.1 g CALB/SCs (72.3 mg g^{-1} of protein), 2 mL *n*-octane containing 1-phenylethanol (0.05 M) and vinyl acetate (0.2 M) were mixed in a 25 mL Schlenk tube, and stirred at 45 °C with a magnetic bar (10 mm in length, 600 rpm). Aliquots of the solution were taken at regular intervals and extracted with diethyl ether to monitor the conversions and e.e. by chiral column equipped GC analysis.

H-beta zeolite-CALB cascade catalytic synthesis of chiral ester in biomimetic microreactor-based flow system. A mixture of 4 mL [BMIM]NTf$_2$, 0.02 g H-beta zeolite, 0.1 g CALB/SCs, 2 mL of *n*-octane, 0.12 g silica emulsifier was stirred at 8000 rpm for 2 min, yielding a biomimetic microreactor. The resultant biomimetic microreactors were gently poured into a glass column reactor (inner diameter of 2 cm) with a sand filter (4.5−9 μm in pore diameter) placed at the bottom of the column. A solution of 1-phenylethanol (0.05 M) and vinyl acetate (0.2 M) in *n*-octane as mobile phase was pumped at a given flow rate through the inlet of the column reactor at the top, and was allowed to pass through the column reactor. The temperature of the column was kept at 45 ºC throughout. The outflow of the column reactor was sampled for GC analysis at regular intervals. The product was further confirmed with GC-MS.

5. Detailed derivation of equations for theoretical investigation.

Definitions

K– Equilibrium constant of reaction (in the absence of enzyme, *i.e.* the second step in cascade reaction)

k_1 – Rate constant of forward reaction for the first step of the cascade reaction

k_2 – Rate constant of backward reaction for the first step of the cascade reaction

k_3 – Rate constant of forward reaction for the second step of the cascade reaction (the backward reaction rate constant k_4 of the second step is safely ignored for the purpose of the calculations, since in our reactions A is overwhelmingly converted to D *via* the forward reaction of the first step, and not the backward reaction of the second step)

A_o^{in} – Initial concentration of reactant A in oil phase

B_o^{in} – Initial concentration of reactant B in oil phase

E_o^{in} – Initial concentration of reactant E in oil phase

α_A – Partition coefficient ($[\text{reagent}]_{\text{IL}}/[\text{reagent}]_{\text{oil}}$) of A

α_B – Diffusion coefficient of B

α_{CD} – partition coefficient of C and D

α_E – partition coefficient of E in IL

ϕ – volume fraction of the IL phase in an emulsion

Derivation

In order to facilitate equation deduction, a model cascade reaction is considered as follows

For step 1, A+B $\rightleftharpoons$(3/5)C +(2/5)D

For step 2, D+E $\rightarrow$ A+F

Note that here the coefficients of proportionality of 3/5 and 2/5 were determined according to the actual reaction considered here. The molecules C and D are chiral mirror images of each other and the factors 3/5 and 2/5 represent a higher chiral bias of the catalyst in producing C relative to D.

(i) Detailed analysis of equilibrium in batch system, and final conversion

The maximum conversion is achieved at equilibrium. To help the analysis of the equilibrium reached in the batch case, we first introduce the following set of parameters for each component. For any component X, we define

$$\beta_X = \alpha_X\phi + (1-\phi) \tag{1}$$

where α_X represents the partition coefficient of the component X between IL and the oil phase. In terms of α_X and $\beta_X$we have the following relations between the concentrations of the component X

in IL phase, in oil and within the whole (total) system:

$$X_{IL} = \alpha_X X_o$$

$$X_{IL} = \frac{\alpha_X}{\beta_X} X_{tot} \qquad (2)$$

$$X_o = \frac{1}{\beta_X} X_{tot}$$

The exchange of the components between different phases in the system occurs much more quickly than the rate at which the reactions proceed. Therefore, it is reasonable to assume that the above relations apply at any time throughout the duration of the reaction. It is easy to check that the average concentration [X] does indeed correspond to X_{tot}, as seen below

$$[X] = \phi X_{IL} + (1-\phi) X_o = \phi \frac{\alpha_X}{\beta_X} X_{tot} + (1-\phi)\frac{1}{\beta_X} X_{tot} = X_{tot} \qquad (3)$$

Now after a sufficiently long time, when equilibrium is attained, the rates of the backward and forward reactions become the same. Hence

$$\frac{3}{5} k_1 B_{IL}^0 A_{IL}(\infty) = \frac{3}{5} k_2 C_{IL}(\infty)$$

$$\frac{2}{5} k_1 B_{IL}^0 A_{IL}(\infty) = \left(\frac{2}{5} k_2 + k_3 E_{IL}^0\right) D_{IL}(\infty) \qquad (4)$$

Once again, as throughout the paper, it is assumed that the reagents B and E are far in excess. This condition is easily satisfied here for our reactions. Hence, one can take the concentrations of B and E as remaining largely constant at their initial values, unaffected by the progress of the reaction. Also, the rate of the backward reaction, converting A to D in the second step is normally far slower than the forward reaction for the same conversion occurring through the first step. As such then, this can be ignored (hence the absence of k_4 in (4)). Expressing all the concentrations in the IL phase in terms of their overall values in the whole system using (2), we have

$$C_{tot}(\infty) = \left(\frac{\beta_{CD}}{\alpha_{CD}}\right)\left(\frac{k_1}{k_2}\right)\left(\frac{\alpha_B}{\beta_B}\right) B_{tot}^0 A_{tot}(\infty)$$

$$D_{tot}(\infty) = \left(\frac{\beta_{CD}}{\alpha_{CD}}\right)\left(\frac{k_1}{k_2 + 2.5 k_3 (\alpha_E/\beta_E) E_{tot}^0}\right)\left(\frac{\alpha_B}{\beta_B}\right) B_{tot}^0 A_{tot}(\infty) \qquad (5)$$

Since every time that an A molecule is consumed it is converted to a C or a D molecule and *vice versa*,

then the total sum of A+C+D in the system is conserved. Furthermore, this should be equal to the initial value of A at the start of the experiment, as introduced within the (1-ϕ) liters of oil, per every liter of emulsion. This is to say that

$$A_{tot}(\infty) + B_{tot}(\infty) + C_{tot}(\infty) = (1-\phi)A_o^{in} \tag{6}$$

Combining equations in (5) with the one above, we arrive at

$$\left[1 + \left(k_1 \frac{\beta_{CD}\alpha_B\alpha_A}{\alpha_{CD}\beta_B\beta_A}\right)\left(\frac{1}{k_2} + \frac{1}{k_2 + 2.5k_3(\alpha_E/\beta_E)E_{tot}^0}\right)B_{tot}^0\right]A_{tot}(\infty) = (1-\phi)A_o^{in}$$

or

$$A_{tot}(\infty) = \left[1 + \left(k_1 \frac{\beta_{CD}\alpha_B\alpha_A}{\alpha_{CD}\beta_B\beta_A}\right)\left(\frac{1}{k_2} + \frac{1}{k_2 + 2.5k_3(\alpha_E/\beta_E)E_{tot}^0}\right)B_{tot}^0\right]^{-1}(1-\phi)A_o^{in} \tag{7}$$

We now recall that the total concentration of B was $B_{tot}^0 = (1-\emptyset)B_o^{in}$ and similarly $E_{tot}^0 = (1-\emptyset)E_o^{in}$. Therefore, equation (7) becomes

$$A_{tot}(\infty) = \left[1 + \left(k_1 \frac{\beta_{CD}\alpha_B\alpha_A}{\alpha_{CD}\beta_B\beta_A}\right)\left(\frac{1}{k_2} + \frac{1}{k_2 + \left[2.5k_3\left(\frac{\alpha_E}{\beta_E}\right)(1-\phi)E_o^{in}\right]}\right)(1-\phi)B_o^{in}\right]^{-1}(1-\phi)A_o^{in} \tag{8}$$

Now the conversion at equilibrium for the batch system can be calculated as

$$\frac{C_{tot}(\infty) + D_{tot}(\infty)}{A_o^{in}(1-\phi)} = 1 - \frac{A_{tot}(\infty)}{A_o^{in}(1-\phi)}$$

$$= 1 - \left[1 + \left(k_1 \frac{\beta_{CD}\alpha_B\alpha_A}{\alpha_{CD}\beta_B\beta_A}\right)\left(\frac{1}{k_2} + \frac{k_1}{k_2 + \left[2.5k_3\left(\frac{\alpha_E}{\beta_E}\right)(1-\phi)E_{tot}^0\right]}\right)(1-\phi)B_o^{in}\right]^{-1} \tag{9}$$

(ii) Detailed analysis of the conversion in a continuous column reactor

For a sufficiently long reaction column, of length L, the steady-state concentrations inside the droplets at the bottom of the column will correspond to their equilibrium values. We once again have

$$\frac{3}{5}k_1 B_{IL}^0 A_{IL}(L) = \frac{3}{5}k_2 C_{IL}(L)$$

$$\frac{2}{5}k_1 B_{IL}^0 A_{IL}(L) = \left(\frac{2}{5}k_2 + k_3 E_{IL}^0\right)D_{IL}(L) \tag{10}$$

where now all the concentrations $X_{IL}(L)$ refer to those at the bottom of the column in the IL phase.

Now let us express these in terms of the concentrations in the oil (at same position at the end of column) using (2). Doing so leads to

$$C_o(L) = \frac{\alpha_A \alpha_B k_1}{\alpha_{CD} k_2} B_o^0 A_o(L)$$

$$D_o(L) = \frac{\alpha_A \alpha_B k_1}{\alpha_{CD}[k_2 + (2.5k_3\alpha_E E_o^0)]} k_1 B_o^0 A_o(L) \tag{11}$$

Note once again that any A converted in the column is changed to C or D and *vice versa*. In a steady-state operation then, the amount of A entering the column through the oil at the inlet should be the same as the total sum of A+C+D in the same amount of oil that is existing the column. Therefore,

$$A_o(L) + C_o(L) + D_o(L) = A_o^{in} \tag{12}$$

Combining the above equation with those in (11) allows one to obtain

$$\left[1 + \left(k_1 \frac{\alpha_B \alpha_A}{\alpha_{CD}}\right)\left(\frac{1}{k_2} + \frac{1}{[k_2 + (2.5k_3\alpha_E E_o^{in})]}\right) B_o^{in}\right] A_o(L) = A_o^{in}$$

or

$$A_o(L) = \left[1 + \left(k_1 \frac{\alpha_B \alpha_A}{\alpha_{CD}}\right)\left(\frac{1}{k_2} + \frac{1}{[k_2 + (2.5k_3\alpha_E E_o^{in})]}\right) B_o^{in}\right]^{-1} A_o^{in} \tag{13}$$

The conversion for the continuous column reactor is then given us

$$\frac{(1-\phi)[C_o(L) + D_o(L)]}{A_o^{in}(1-\phi)} = 1 - \frac{A_o(L)}{A_o^{in}}$$

$$= 1 - \left[1 + \left(k_1 \frac{\alpha_B \alpha_A}{\alpha_{CD}}\right)\left(\frac{1}{k_2} + \frac{1}{[k_2 + (2.5k_3\alpha_E E_o^{in})]}\right) B_o^{in}\right]^{-1} \tag{14}$$

(iii) Detailed analysis of the cascade reaction on the length scale of a single droplet

The diffusion-reaction equations governing the evolution of the concentration of reactants and products, for the model two step cascade reaction introduced above, are as follows within a droplet:

$$\frac{1}{d_f}\frac{\partial A}{\partial t} = \nabla^2 A - (\omega_1 A) + \left(\frac{3}{5}\omega_2 C\right) + \left(\left[\frac{2}{5}\omega_2 + \omega_3\right] D\right) \tag{15}$$

$$\frac{1}{d_f}\frac{\partial C}{\partial t} = \nabla^2 C + \left(\frac{3}{5}\omega_1 A\right) - \left(\frac{3}{5}\omega_2 C\right) \tag{16}$$

$$\frac{1}{d_f}\frac{\partial D}{\partial t}=\nabla^2 D+\left(\frac{2}{5}\omega_1 A\right)-\left(\left[\frac{2}{5}\omega_2+\omega_3\right]D\right) \quad . \tag{17}$$

In the above equations d_f is the diffusion coefficient, assumed to be the same for A, C and D, in the IL phase. We also define symbols

$$\begin{aligned}\omega_1 &= k_1 B_{IL}^0 / d_f \\ \omega_2 &= k_2 / d_f \\ \omega_3 &= k_3 E_{IL}^0 / d_f\end{aligned} \tag{18}$$

where, as mentioned above, the concentration of B and E are assumed to be far in excess of C, D and A. Therefore they are considered to remain approximately constant at their initial values B_{IL}^0 and E_{IL}^0 everywhere inside the droplet throughout the process.

Under steady state conditions, the concentration of the components stabilizes and ceases to vary with time. Under such circumstances, the above equations reduce to

$$\nabla^2 A=\left(\omega_1 A\right)-\left(\frac{3}{5}\omega_2 C\right)-\left(\left[\frac{2}{5}\omega_2+\omega_3\right]D\right) \tag{19}$$

$$\nabla^2 C=-\left(\frac{3}{5}\omega_1 A\right)+\left(\frac{3}{5}\omega_2 C\right) \tag{20}$$

$$\nabla^2 D=-\left(\frac{2}{5}\omega_1 A\right)+\left(\left[\frac{2}{5}\omega_2+\omega_3\right]D\right) \quad . \tag{21}$$

Now we add the above three equations together to obtain

$$\nabla^2(A+C+D)=0$$

which has the general solution

$$(A+C+D)=\frac{Q}{r}+q$$

within the droplet. Since the total concentration (A+C+D) is finite at the centre of the droplet (r = 0), this implies that Q = 0. Therefore, the total sum of the concentration of the three components A, C and D, remains uniform inside the droplet, equal to (as yet undetermined) constant q. In other words

$$A = q - (C + D) \quad . \quad (22)$$

We can use the above result to eliminate A from equations (20) and (21) in favour of C and D. Doing so leads to

$$\nabla^2 C = -\left(\frac{3}{5}\omega_1 q\right) + \left(\frac{3}{5}(\omega_1 + \omega_2)\right)C + \left(\frac{3}{5}\omega_1\right)D \quad (23a)$$

and

$$\nabla^2 D = -\left(\frac{2}{5}\omega_1 q\right) + \left(\frac{2}{5}\omega_1\right)C + \left(\left[\frac{2}{5}(\omega_1 + \omega_2) + \omega_3\right]\right)D \quad (23b)$$

which more conveniently can be represented in a matrix form $\nabla^2\mathbf{u}=(\mathbf{Mu})-\mathbf{v}$, with the 2 X 2 matrix $\mathbf{M}$

$$\mathbf{M} = \begin{pmatrix} \frac{3}{5}(\omega_1 + \omega_2) & \frac{3}{5}\omega_1 \\ \frac{2}{5}\omega_1 & \omega_3 + \frac{2}{5}(\omega_1 + \omega_2) \end{pmatrix} \quad . \quad (24)$$

and vectors $\mathbf{u}$ and $\mathbf{v}$ defined as

$$\mathbf{u} = \begin{pmatrix} C \\ D \end{pmatrix} \quad \text{and} \quad \mathbf{v} = (\omega_1 q / 5)\begin{pmatrix} 3 \\ 2 \end{pmatrix} \quad . \quad (25)$$

The pair of equations in (23) are coupled. In order to solve them one needs to manipulate these so as to obtain two decoupled equations. This can be done by diagonalising $\mathbf{M}$, by first calculating the two eigenvalues, and the corresponding eigenvectors of its transpose matrix, $\mathbf{M}^\mathbf{T}$. The eigenvalues $\Lambda^\pm$ are given as usual by solving the equation $(m_{11}-\Lambda)(m_{22}-\Lambda) - m_{12}\, m_{21} = 0$, where m_{ij} refers to the ijth element of the matrix $\mathbf{M}^\mathbf{T}$. The solutions to this equation are

$$\Lambda^\pm = \frac{S \pm S\sqrt{1-H}}{2}$$

where for abbreviation we have defined

$$S = \omega_1 + \omega_2 + \omega_3 \quad (26)$$

and

$$H=\frac{24}{25S^2}\left((S-\omega_3)(S+\frac{3}{2}\omega_3)-\omega_1^2\right) \quad . \tag{27}$$

Denoting the corresponding eigenvectors as $\mathbf{x}_+$ and $\mathbf{x}_-$, these are calculated to be

$$\begin{aligned}\mathbf{x}_+^T&=\left(\frac{1}{6}\frac{S}{\omega_1}-\frac{\omega_3}{\omega_1}+\frac{5}{6}\frac{S}{\omega_1}\sqrt{(1-H)}\ ,\ 1\right)\\ \mathbf{x}_-^T&=\left(\frac{1}{6}\frac{S}{\omega_1}-\frac{\omega_3}{\omega_1}-\frac{5}{6}\frac{S}{\omega_1}\sqrt{(1-H)}\ ,\ 1\right)\end{aligned} \quad . \tag{28}$$

Now, by forming the matrix $\begin{pmatrix}\mathbf{x}_+^T\\ \mathbf{x}_-^T\end{pmatrix}$ and multiplying both sides of the equation $\nabla^2\mathbf{u}=(\mathbf{Mu})-\mathbf{v}$ by this matrix, we arrive at

$$\nabla^2\begin{pmatrix}h_1\\ h_2\end{pmatrix}=\begin{pmatrix}\Lambda_+h_1\\ \Lambda_-h_2\end{pmatrix}-(q/10)\begin{pmatrix}4\omega_1+S-6\omega_3+5S\sqrt{(1-H)}\\ 4\omega_1+S-6\omega_3-5S\sqrt{(1-H)}\end{pmatrix} \quad , \tag{29}$$

with functions h_1 and h_2 formed from linear combinations of C and D according to:

$$h_1=D+\left[\frac{S-6\omega_3+5S\sqrt{(1-H)}}{6\omega_1}\right]C \tag{30a}$$

$$h_2=D+\left[\frac{S-6\omega_3-5S\sqrt{(1-H)}}{6\omega_1}\right]C \quad . \tag{30b}$$

It should be noted that the two equations for h_1 and h_2 in (29) are now completely decoupled from each other, and thus can be solved separately. In particular, once h_1 and h_2 are determined, the required concentrations of C and D inside the droplet are calculated from

$$C=\frac{3\omega_1}{5S\sqrt{1-H}}\left(h_1-h_2\right) \tag{31a}$$

and

$$D = \frac{1}{10\sqrt{1-H}}\left(1 - \frac{6\omega_3}{S}\right)(h_2 - h_1) \; + \; \frac{1}{2}(h_2 + h_1)$$
$$= \frac{1}{2}(h_2 + h_1) + \frac{(6\omega_3 - S)}{6\omega_1}C \quad . \qquad (31b)$$

To solve equation (29) we express the ∇^2 operator in their polar spherical coordinates. Taking advantage of the spherical symmetry of the droplet, the decoupled set of equations for h_1 and h_2 in this coordinate system now read

$$\frac{\partial^2 (rh_1)}{dr^2} = \Lambda^+ (rh_1) - q\varepsilon_1 r \qquad (32a)$$

and

$$\frac{\partial^2 (rh_2)}{dr^2} = \Lambda^- (rh_2) - q\varepsilon_2 r \quad , \qquad (32b)$$

where, as seen from equation (29), constants ε_1 and ε_2 are

$$\varepsilon_1 = \frac{4\omega_1 + S - 6\omega_3 + 5S\sqrt{(1-H)}}{10} \qquad (33a)$$

and

$$\varepsilon_2 = \frac{4\omega_1 + S - 6\omega_3 - 5S\sqrt{(1-H)}}{10} \quad . \qquad (33b)$$

The solution to equations (32a) and (32b) can readily be obtained and read

$$h_1 = \frac{a_1}{r}\sinh(r/\zeta_1) + \zeta_1^2 q\varepsilon_1$$

(34a)

$$h_2 = \frac{a_2}{r}\sinh(r/\zeta_2) + \zeta_2^2 q\varepsilon_2 \qquad (34b)$$

with the two constants of integration a_1 and a_2 to be determined, together with q, from the boundary conditions at the oil-droplet interface (as well as those as r Ý ∞), as discussed later on. Equations (34a) and (34b), in conjunction with (31), indicate that the concentrations of both C and D are governed by two distinct length scales, ζ_1 and ζ_2, as given by

$$\zeta_1 = \frac{1}{\sqrt{\Lambda^+}} = \left[\frac{(\omega_1+\omega_2+\omega_3)}{2}\left(1+\sqrt{1-\frac{24}{25(\omega_1+\omega_2+\omega_3)^2}\left((\omega_1+\omega_2)(\omega_1+\omega_2+\frac{5}{2}\omega_3)-\omega_1^2\right)}\right)\right]^{-1/2} \quad (35a)$$

and

$$\zeta_2 = \frac{1}{\sqrt{\Lambda^-}} = \left[\frac{(\omega_1+\omega_2+\omega_3)}{2}\left(1-\sqrt{1-\frac{24}{25(\omega_1+\omega_2+\omega_3)^2}\left((\omega_1+\omega_2)(\omega_1+\omega_2+\frac{5}{2}\omega_3)-\omega_1^2\right)}\right)\right]^{-1/2} \quad (35b)$$

Next we consider the diffusion in the oil phase. It is assumed that no (or extremely slow) reactions occur in oil, since no catalyst or enzyme is present there. Therefore, the mass transport equations in this phase, once the steady state has been achieved, simply become ∇^2A=0, ∇^2C=0 and ∇^2D=0. Solved once again in the spherical polar coordinates, the solution to these equations are

$$A(r) = A_O^0 - \frac{\gamma_A}{r} \quad , \quad (36a)$$

$$C(r) = C_O^0 + \frac{\gamma_C}{r} \quad (36b)$$

and

$$D(r) = D_O^0 + \frac{\gamma_D}{r} \quad , \quad (36c)$$

where A_o^0, C_o^0 and D_o^0 are the concentrations of A, C and D, respectively, in the oil phase at a point far from the droplet. The parameters γ_A, γ_C and γ_D are the constants of integration which again need to be obtained by matching the concentrations of various components at the IL-oil interface, as is discussed below.

The six unknown constants of integration a_1, a_2, q, γ_A, γ_C and γ_D, resulting in the solution of the diffusion-reaction equations inside the droplet and those outside, can be determined from the six equations on the surface of droplets. The concentration of each component at the two sides of the IL-oil boundary have to be in equilibrium with each other, and there should also be no discontinuities in the flux of each component across the interface. Written down explicitly, and using (22), (31), (34) and (36), we have

$$\alpha_C \frac{\gamma_C}{R} + \alpha_C C_o^0 = \frac{3\omega_1}{5S\sqrt{1-H}}\left(\frac{a_1}{R}\sinh(R/\zeta_1) - \frac{a_2}{R}\sinh(R/\zeta_2) + \zeta_1^2 q\varepsilon_1 - \zeta_2^2 q\varepsilon_2\right) \tag{37a}$$

$$\alpha_D \frac{\gamma_D}{R} + \alpha_D D_o^0 = \frac{1}{2}(h_2 + h_1) + \frac{(6\omega_3 - S)}{6\omega_1} C =$$
$$\frac{1}{2}\left(\frac{a_2}{R}\sinh(R/\zeta_2) + \frac{a_1}{R}\sinh(R/\zeta_1) + \zeta_2^2 q\varepsilon_2 + \zeta_1^2 q\varepsilon_1\right) + \frac{(6\omega_3 - S)}{6\omega_1}\left(\alpha_C \frac{\gamma_C}{R}\right) \tag{37b}$$

$$\alpha_A\left(A_0^0 - \frac{\gamma_A}{R}\right) = \left(q - C - D\right) = q - \alpha_C \frac{\gamma_C}{R} - \alpha_D \frac{\gamma_D}{R} - \alpha_C C_o^0 - \alpha_D D_0^0 \tag{37c}$$

where as before we have denoted the partition coefficient of A, C and D as α_A , α_C , and α_D . For the continuity of the fluxes we have

$$\left.\frac{\partial A}{\partial r}\right|_{R^-} = \left(\frac{d_f^o}{d_f}\right)\left.\frac{\partial A}{\partial r}\right|_{R^+}$$

$$\left.\frac{\partial C}{\partial r}\right|_{R^-} = \left(\frac{d_f^o}{d_f}\right)\left.\frac{\partial C}{\partial r}\right|_{R^+}$$

and

$$\left.\frac{\partial D}{\partial r}\right|_{R^-} = \left(\frac{d_f^o}{d_f}\right)\left.\frac{\partial D}{\partial r}\right|_{R^+}$$

To distinguish the diffusion coefficient in the oil from that in IL, we use the symbol d_f^o for the former phase. When expressed explicitly, the above equations become

$$-\frac{d_f^o}{d_f}\left(\frac{\gamma_C}{R^2}\right) = \frac{3\omega_1}{5S\sqrt{1-H}}\frac{1}{R^2}\left(a_1\frac{R}{\zeta_1}\cosh(R/\zeta_1) - a_1\sinh(R/\zeta_1) - a_2\frac{R}{\zeta_2}\sinh(R/\zeta_2) + a_2\sinh(R/\zeta_2)\right),$$

$$-\frac{d_f^o}{d_f}\left(\frac{\gamma_D}{R^2}\right) = \frac{1}{2R^2}\left(a_2\frac{R}{\zeta_2}\cosh(R/\zeta_2) - a_2\sinh(R/\zeta_2) + a_1\frac{R}{\zeta_1}\cosh(R/\zeta_1) - a_1\sinh(R/\zeta_1)\right)$$
$$-\frac{(6\omega_3 - S)}{6\omega_1}\left(\frac{d_f^o}{d_f}\right)\left(\frac{\gamma_C}{R^2}\right)$$

and

$$\frac{d_f^o}{d_f}\left(\frac{\gamma_A}{R^2}\right)=\frac{d_f^o}{d_f}\left(\frac{\gamma_C}{R^2}\right)+\frac{d_f^o}{d_f}\left(\frac{\gamma_D}{R^2}\right) \tag{38}$$

These can further be simplified to

$$\gamma_C=\frac{-3\omega_1}{5S\sqrt{1-H}}\left(\frac{d_f}{d_f^o}\right)\left(a_1\frac{R}{\zeta_1}\cosh(R/\zeta_1)-a_1\sinh(R/\zeta_1)-a_2\frac{R}{\zeta_2}\sinh(R/\zeta_2)+a_2\sinh(R/\zeta_2)\right), \tag{39a}$$

$$\gamma_D=-\frac{1}{2}\left(\frac{d_f}{d_f^o}\right)\left(a_2\frac{R}{\zeta_2}\cosh(R/\zeta_2)-a_2\sinh(R/\zeta_2)+a_1\frac{R}{\zeta_1}\cosh(R/\zeta_1)-a_1\sinh(R/\zeta_1)\right)+\frac{(6\omega_3-S)}{6\omega_1}\gamma_C \tag{39b}$$

and

$$\gamma_A=\gamma_C+\gamma_D \quad . \tag{39c}$$

To solve the set of simultaneous equations (37) and (39), first consider equation (37c) together with equation (39c), so as to express variable q in the following form

$$Rq=R\left(\alpha_A A_o^0+\alpha_C C_o^0+\alpha_D D_o^0\right)+\left(\alpha_C-\alpha_A\right)\gamma_C+\left(\alpha_D-\alpha_A\right)\gamma_D \tag{40}$$

Next we re-write the first two equations in (37) as

$$\gamma_C=\frac{Z}{\alpha_C}\left(a_1\sinh(R/\zeta_1)-a_2\sinh(R/\zeta_2)\right)+Rq\frac{Z}{\alpha_C}(\zeta_1^2\varepsilon_1-\zeta_2^2\varepsilon_2)-RC_o^0 \tag{41a}$$

and

$$\gamma_D=\frac{1}{2\alpha_D}\left(a_2\sinh(R/\zeta_2)+a_1\sinh(R/\zeta_1)\right)+ Rq\frac{1}{2\alpha_D}(\zeta_2^2\varepsilon_2+\zeta_1^2\varepsilon_1)+Y\left(\frac{\alpha_C}{\alpha_D}\right)\left(\gamma_C+RC_o^0\right)-RD_o^0 \tag{41b}$$

where to simply the expressions we have defined

$$Y=\frac{(6\omega_3-S)}{6\omega_1} \quad \text{and} \quad Z=\frac{3\omega_1}{5S\sqrt{1-H}}$$

Similarly, in terms of Y and Z, equations (39a) and (39b) read

$$\gamma_C = a_2 Z\left(\frac{d_f}{d_f^o}\right)\left(\frac{R}{\zeta_2}\cosh(R/\zeta_2) - \sinh(R/\zeta_2)\right) - a_1 Z\left(\frac{d_f}{d_f^o}\right)\left(\frac{R}{\zeta_1}\cosh(R/\zeta_1) - \sinh(R/\zeta_1)\right) \tag{42a}$$

and

$$\gamma_D = -a_2\left(\frac{d_f}{2d_f^o}\right)\left(\frac{R}{\zeta_2}\cosh(R/\zeta_2) - \sinh(R/\zeta_2)\right)$$
$$- a_1\left(\frac{d_f}{2d_f^o}\right)\left(\frac{R}{\zeta_1}\cosh(R/\zeta_1) - \sinh(R/\zeta_1)\right) + Y\gamma_C$$

which further is expressed as

$$\begin{aligned}\gamma_D = a_2\left(\frac{d_f}{2d_f^o}\right)(YZ-1)\left(\frac{R}{\zeta_2}\cosh(R/\zeta_2) - \sinh(R/\zeta_2)\right)\\ - a_1\left(\frac{d_f}{2d_f^o}\right)(YZ+1)\left(\frac{R}{\zeta_1}\cosh(R/\zeta_1) - \sinh(R/\zeta_1)\right)\end{aligned} \tag{42b}$$

Equations (42a) and (42b) fully express γ_C and γ_D, and therefore also q and γ_A viz. equations (40) and (39c), in terms of the other two remaining variables a_1, a_2. When these are substituted into equations (41a) and (41b), we end up with just two simultaneous linear equations, involving the two unknown variables a_1, a_2 to solve. It is now a simple matter to obtain the values of a_1, a_2 and hence also γ_C, γ_D, γ_A and q from (42a), (42b), (39c) and (40), by solving these two equations. The resulting expressions for these variables, though simple to derive, are rather lengthy and so are not reproduced explicitly here. More conveniently the actual numerical values of a_1, a_2 are calculated in a simple spreadsheet for all systems of interest.

Supplementary Mass Spectrometry Data

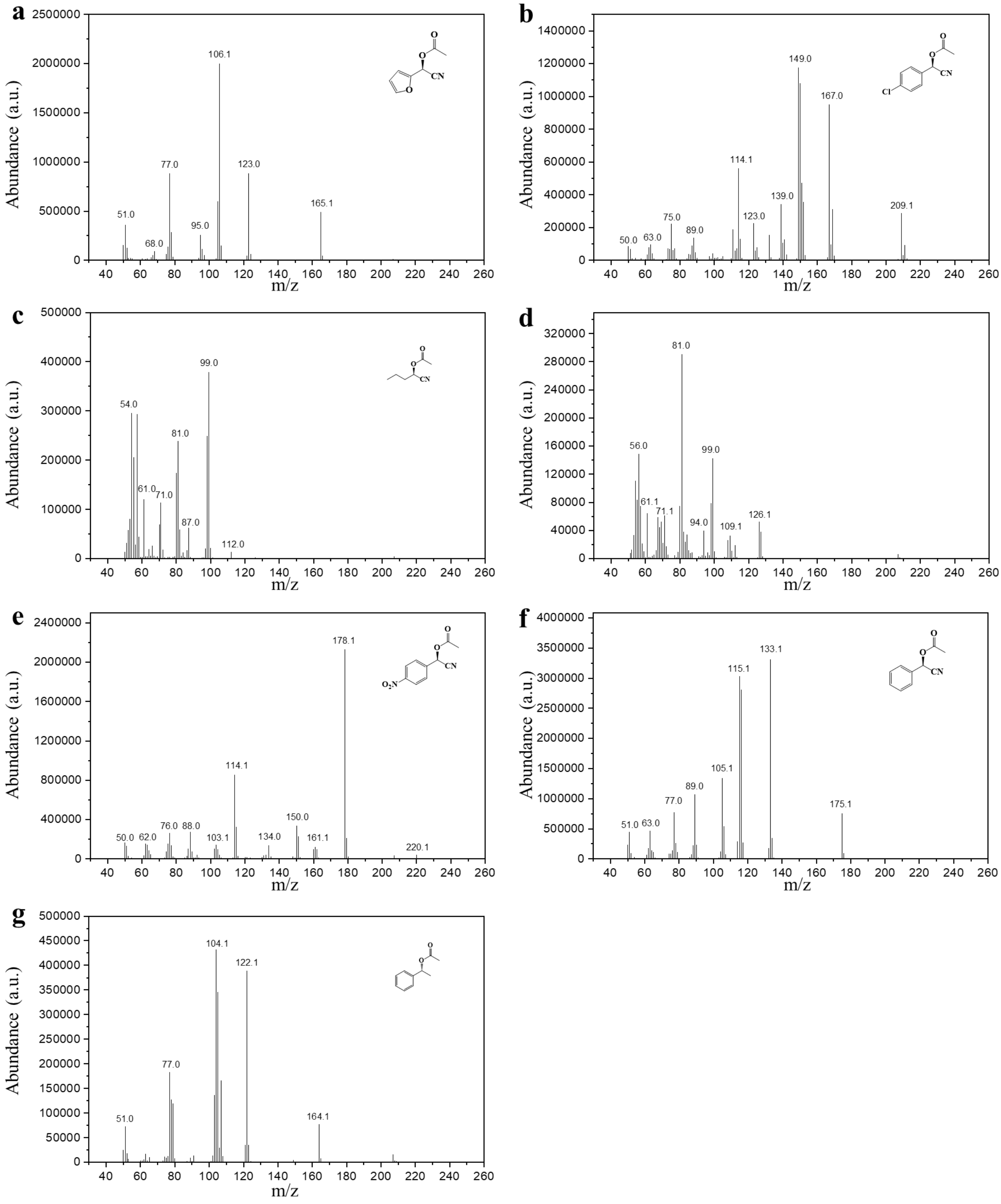

Supplementary data 1. Mass spectrometry spectra for chiral products. a-f, O-acylated cyanohydrins synthesized through Ti(Salen)-CALB cascade catalysis. **g**, 1-phenylethanol acetate synthesized through H-beta zeolite-CALB cascade catalysis.

Supplementary References

1. Shen, D. K. et al. Biphase stratification approach to three-dimensional dendritic biodegradable mesoporous silica nanospheres. *Nano Lett*. **14**, 923−932, (2014).

2. Belokon, Y. N. et al. The asymmetric addition of trimethylsilyl cyanide to aldehydes catalyzed by chiral (Salen) titanium complexes. *J. Am. Chem. Soc.* **121**, 3968−3973, (1999).

3. Kong, D. Y. et al. Tunable photoluminescence in monodisperse silica spheres. *J. Colloid Interface Sci*. 352, 278−284, (2010).
